# Mapping the dialogue: Decoding alveolar stem–niche interactions

**DOI:** 10.1073/pnas.2606113123

**Published:** 2026-07-08

**Authors:** Ahmad N. Nabhan, Anne Biton, Christine Everett, Conrad Foo, Diana Wu, Joshua D. Webster, Alina A. Alam, Elisa Penna, Sandra Rost, Neha Rohatgi, Rohit Reja, Ranel J. Tulpano, Shiqi Xie, Celine Eidenschenk, Kim Newton, Joseph R. Arron, Vishva M. Dixit

**Affiliations:** ^a^ Department of Molecular and Cellular Biology, University of California, Berkeley, CA 94720; ^b^Department of Physiological Chemistry, Genentech, South San Francisco, CA 94080; ^c^Computational Sciences, Genentech, South San Francisco, CA 94080; ^d^Department of Molecular Discovery and Cancer Cell Biology, Genentech, South San Francisco, CA 94080; ^e^Department of Pathology, Genentech, South San Francisco, CA 94080; ^f^Roche Informatics, Hoffman-La Roche Canada, Mississauga, ON, Canada; ^g^Department of Immunology, Genentech, South San Francisco, CA 94080

**Keywords:** lung, epithelium, fibroblast, Nkx2.1, niche

## Abstract

While cellular atlases have revealed remarkable phenotypic diversity, how cells navigate this landscape to influence tissue behavior remains poorly understood. We present an alveolosphere screening platform for investigating interactions between lung stem cells and their fibroblast niche. We assessed the role of 201 candidate genes in stem cells via imaging, then used chimeric RNAseq analysis for a transcriptome-wide understanding of cell-autonomous effects on stem cells and non-cell-autonomous effects on the niche. This phenome-transcriptome map uncovered cellular states and pathways regulating proliferation, metabolism, and immune signaling. Notably, stem cells influenced scar-forming and immune programs in fibroblasts. This injury response was dependent on stem cell identity; loss of *Nkx2.1*, encoding the transcription factor conferring lung epithelial identity, rewired stem cell–niche interactions and had a greater non-cell-autonomous effect than eliminating the cancer genes *Trp53, Egfr,* or *Cdkn2b*. Our study highlights how functional atlases complement the cellular diversity revealed by descriptive methods.

## Significance

Alveolar Type 2 (AT2) epithelial cells and their surrounding fibroblast niche collaborate to preserve the lung’s gas-exchange surface. When this partnership breaks down, it contributes to lung adenocarcinoma and degenerative lung diseases. We conducted a large-scale, multimodal genetic screen focused on AT2 cell biology. This approach systematically identified the receptors and signaling pathways required for AT2 cell proliferation and secretory function. Importantly, we also examined how alterations in AT2 cells reshape their fibroblast niche—an interaction central to tumor microenvironments and fibrotic remodeling but difficult to dissect with traditional methods. Our findings reveal an unexpected role for AT2 cells in directing fibroblast transcriptional programs that influence fibrosis, immune cell trafficking, and metabolic regulation.

The molecular crosstalk between epithelial stem cells and their stromal niches drives tissue homeostasis and injury repair (1, 2). One of the simplest stem–niche systems occurs in the lung alveolar epithelium. This delicate barrier tissue contains flat AT1 cells for gas diffusion (3) and cuboidal Alveolar Type 2 (AT2) cells that secrete surfactants to prevent alveolar collapse. AT2 cells also serve as stem cells, self-renewing intermittently and giving rise to AT1 cells (4, 5). AT2 cells are abutted by alveolar fibroblasts, which act as a single cell niche providing signals required for AT2 stem cell activity (4, 6). Dysfunction of this stem–niche system underlies lung adenocarcinoma (5, 7) and pulmonary fibrosis (8–10), and can impact viral diseases such as COVID-19 (11, 12).

While single-cell atlases and human genetic studies (13–17) have identified hundreds of genes that may play a role in alveolar stem–niche communication, a detailed mechanistic understanding of their functions remains elusive. Studying each new gene has relied on labor-intensive engineered mouse models. Pooled genetic screens in cell lines allow greater throughput but sacrifice physiological fidelity and the cellular complexity for studying intercellular signaling. Organoids recapitulate in vivo tissue biology better than cell lines, while maintaining the throughput and accessibility of in vitro systems (18, 19). The alveolosphere organoid system combines primary AT2 cells with lung fibroblasts at an air–liquid interface. Signals from fibroblasts drive AT2 cell proliferation. Subsequently, a subset of the AT2 cells differentiate into gas-exchanging AT1 cells (4). This model preserves intercellular signaling, enabling studies of niche–stem crosstalk. By contrast, other lung, liver, intestine, and kidney organoid models include only epithelial components (19–21). Models derived from induced pluripotent stem cells, like those for the brain (22) or kidney (23), fall at the other end of the spectrum as they involve numerous cell types and cellular communication is difficult to deconvolve. The disadvantages of the alveolosphere system are that phenotyping typically utilizes low-content imaging and omics analyses require sorting of stem cells from the niche.

We developed a miniaturized chimeric mouse AT2 cell and human fibroblast alveolosphere assay with CRISPR knockout (KO) to study AT2 cell-fibroblast crosstalk. Testing 201 genes via a multimodal readout, we identified regulators of AT2 stem cells and potential therapeutic targets that modulate their secretory, immune, and stem properties. Transcriptomics revealed both previously described and additional AT2 cell transcriptional states, including diverging trajectories within the Krt8^+^ state previously associated with alveolar regeneration after lung injury (24–26). Gene KOs in AT2 cells were also found to impact proinflammatory, metabolic, and fibrotic processes in fibroblasts. These non-cell-autonomous effects were correlated with AT2 cell proliferation, implying stem cell coordination of tissue repair. For example, deleting *Nkx2.1* in AT2 cells had a non-cell-autonomous effect on fibroblast identity. Spatial transcriptomics identified similar stromal remodeling after *Nkx2.1* KO in vivo. Collectively, our findings offer a functional roadmap of pathways that define lung alveolar stem cell identity, metabolism, and proliferation, as well as those influencing the fibroblast niche.

## Results

### A Chimeric Platform for Dissecting Stem–Niche Crosstalk.

**Fig. 1.**
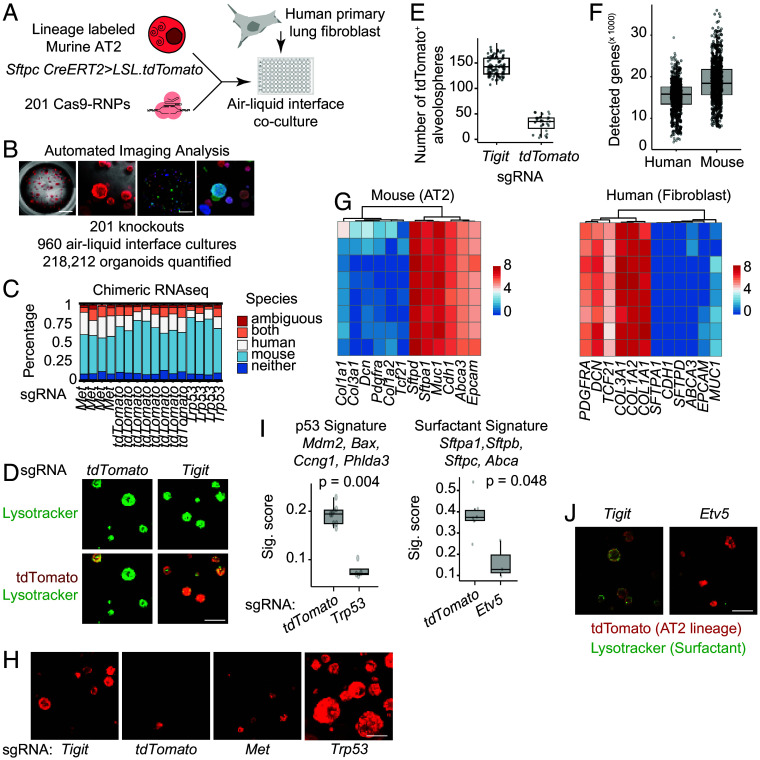
Multimodal genetic alveolosphere screen for decoding stem–niche communication. (*A*) Chimeric alveolosphere scheme. (*B*) tdTomato^+^ alveolospheres (*Left*) and their segmentation masks (*Right*). (Scale bar, 1,000 μm.) (*C*) Percentage of alveolosphere RNAseq reads identified as human or mouse. Bars, individual wells. (*D*) Expression of AT2 lineage marker tdTomato (red) in alveolospheres stained with lysotracker (green). (Scale bar, 500 μm.) (*E*) Numbers of tdTomato^+^ alveolospheres per well after deletion of *tdTomato* (n = 31 wells) or *Tigit* (n = 91 wells). (*F*) The number of mouse and human genes detected in chimeric alveolosphere cultures (n = 793 wells). (*G*) Heatmaps indicate expression [log2 count per million (CPM)] of AT2 and fibroblast genes in the mouse (*Left*) and human (*Right*) transcriptomes of chimeric alveolospheres targeted with *tdTomato*^sgRNA^ (n = 8 wells). (*H*) Alveolospheres after CRISPR KO of the indicated genes in AT2 cells. (Scale bar, 500 μm.) (*I*) Expression of the indicated gene signatures (*Materials and Methods*) in alveolospheres grown using AT2 cells lacking *Trp53* (n = 4 wells), *Etv5* (n = 4 wells), or *tdTomato* (n = 8 wells). *P*-values determined by the two-sided Student *t* test. (*J*) tdTomato^+^ alveolospheres targeted with sgRNAs against *Tigit* (control) or *Etv5* stained with Lysotracker.

To investigate interactions between AT2 cells and fibroblasts, we combined Cas9-mediated gene KO in AT2 cells with a chimeric alveolosphere assay (Fig. 1 *A*–*C*). In an arrayed format (Dataset S1), mouse tdTomato-expressing AT2 cells were electroporated with Cas9 ribonucleotide particles (RNP) carrying three distinct sgRNAs for each gene (Dataset S2) and then cultured with human lung fibroblasts to form organoids. sgRNAs targeting the *Rosa26.tdTomato* reporter and *Tigit*, the latter not expressed in AT2 cells, served as positive and negative controls, respectively. The number of tdTomato^+^ alveolospheres in positive and negative control wells suggested that our gene KO efficiency was approximately 80% (Fig. 1 *D* and *E*). Wells were imaged after 7 and 14 d of culture. Alveolosphere numbers and sizes were determined with “Segment Anything” (Fig. 1*B*). Chimeric alveolospheres in control wells had a dense morphology with proliferating Ki67^+^ Muc1^+^ SftpC^+^ AT2 cells at the periphery and Rage^+^ Hopx^+^ AT1 cells closer to the lumen (*SI Appendix*, Fig. S1 *A*–*C*). Alveolospheres composed of only mouse AT2 cells have a similar organization (4). Chimeric alveolospheres in control wells were labeled by Lysotracker (Fig. 1*D*), a dye that accumulates in the surfactant-storing lamellar bodies of AT2 cells.

Full-length Smart-seq3 cDNA libraries were prepared using RNA from day 14 alveolospheres. Sequencing yielded approximately 5 million reads per library and these were assigned to the mouse and human genomes for independent analysis. Although we seeded mouse AT2 cells and human fibroblasts at a ratio of 1:10, approximately 60% of reads aligned with the mouse genome and 20% with the human genome (Fig. 1*C*). This result is consistent with AT2 cells proliferating more than fibroblasts in the organoids. Accordingly, the proportion of mouse reads scaled with the alveolosphere number per well (*SI Appendix*, Fig. S2). On average, we detected 20,000 genes in the mouse transcriptome and 18,000 in the human transcriptome (Fig. 1*F*). The mouse transcriptome was enriched for AT2 and epithelial markers by several orders of magnitude, and the human transcriptome for fibroblast markers (Fig. 1*G*), indicating the successful capture of cell-type specific transcriptomes. Reads that aligned to both transcriptomes (11%) and those that were ambiguous (3%) were excluded from subsequent analyses.

We selected 201 genes for KO in the AT2 cells of chimeric alveolospheres (Dataset S2). Most were implicated in alveolar biology by genome-wide association studies or single cell transcriptomics but had ill-defined functions. A smaller subset had known cell autonomous roles in alveolar epithelial cells and were used to validate our screening approach. For example, deletion of *Met*, which is essential for the proliferation or survival of AT2 cells in mice (27), suppressed alveolosphere growth, whereas loss of the tumor suppressor gene *Trp53* (encoding p53) produced larger alveolospheres (Fig. 1*H*). Transcriptomic analysis confirmed that *Trp53* deficiency reduced the expression of p53 target genes, including *Mdm2* and *Bax* (Fig. 1*I*). Deletion of *Etv5*, a transcription factor gene required for surfactant production (28), reduced the expression of surfactant genes, including *Sftpb* and *Sftpc* (Fig. 1*I*), and yielded alveolospheres that stained poorly with lysotracker (Fig. 1*J*). Thus, gene deletion in chimeric alveolospheres can recapitulate known lung phenotypes.

We compared the total expression level for genes that had sufficient coverage [log2 counts per million (CPM) > 4, 123 out of 201 genes]. Differential expression analysis showed that 72% had significantly reduced expression (*SI Appendix*, Fig. S3*A*), which is comparable to methodologies employing viral transduction and selection techniques (29, 30). For a subset of the wells (15.3%), the regions targeted by sgRNAs had sufficient sequencing coverage to quantify gene editing events such as indels, cryptic junctions, and single nucleotide polymorphisms (SNPs). Of these, 77.7% (91/117) appeared to be edited. For the remaining 84.7%, coverage was insufficient to determine editing efficiency (for example, *Ryk*; *SI Appendix*, Fig. S3*B*). We often observed localized deletion over the amplicon site (for example, *Xbp1;* *SI Appendix*, Fig. S3*C*). For *Nkx2.1,* we observed evidence of gene editing but an increase in gene expression. However, immunolabeling demonstrated a loss of Nkx2.1 protein (*SI Appendix*, Fig. S3*D*). These data confirm the platform is suited to interrogating the role of specific genes in niche–stem cell signaling.

### Identifying Regulators of AT2 Stem Cell Activity.

**Fig. 2.**
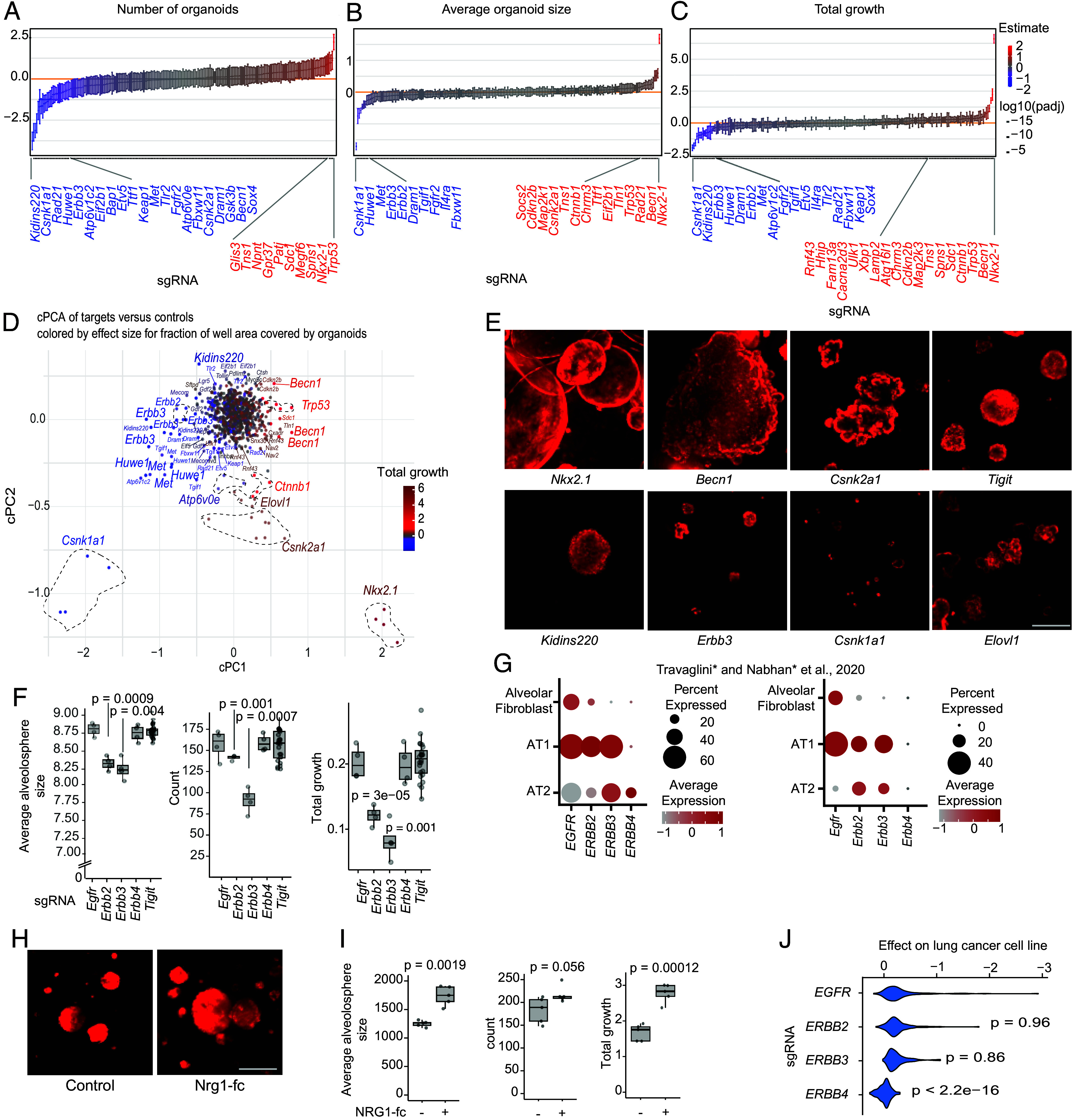
Identification of pathways promoting AT2 cell proliferation and alveolosphere growth. (*A*–*C*) The number (*A*), average size (*B*), and total growth (*C*; fraction of well covered by segmented regions) of alveolospheres for each KO relative to the *Tigit* sgRNA in-plate controls. sgRNAs increasing these parameters significantly are red. sgRNAs decreasing them are blue. Error bars, 95% CI. Points represent effect size estimates (linear model coefficients). The significance of effects across all three parameters was determined using *t* tests on the coefficients from linear regression models. *P*-values were adjusted using the Benjamini–Hochberg false discovery rate (FDR) correction. (*D*) cPCA of the Segment-Anything embeddings from segmented alveolosphere regions, averaged per well. Each dot represents a well with the specified gene KO, plotted in the space defined by cPC1 and cPC2. The color of each dot represents the effect size of the difference in the fraction of the well covered by alveolospheres between KO and control samples. (*E*) tdTomato^+^ alveolospheres from (*A*–*C*). Results representative of four replicates per sgRNA. (Scale bar, 500 μm.) (*F*) The average size (*Left*), number (*Center*), and total growth (*Right*) of alveolospheres for four KOs: *Egfr, Erbb2, Erbb3, Erbb4* (n = 4 replicates per gene) as well as the in-plate *Tigit* sgRNA control (n = 24). (*G*) The mean level of gene expression (dot intensity, red scale) and the percentage of positive cells (dot size) determined by single cell RNAseq of human (*Left*) and mouse (*Right*) lungs. (*H*) tdTomato^+^ alveolospheres cultured with PBS vehicle (*Left*) or Nrg1-fc (*Right*). Results representative of five replicates per condition. (Scale bar, 500 μm.) (*I*) The size (*Left*), number (*Center*), and total growth (*Right*) of the alveolospheres in *H*. (*J*) Violin plots indicate the fitness scores (*Materials and Methods*) of 96 non–small cell lung adenocarcinoma cell lines following KO of the indicated receptors. *P*-values determined by one-way ANOVA with multiple hypothesis correction (*F* and *J*) or by the Student *t* test (*I*).

We compared the number, average size, and overall growth (fraction of well area covered by organoids) of each gene KO against the in-plate *Tigit* sgRNA controls. 25 of the 201 targeted genes significantly influenced one or more of these parameters (Fig. 2 *A*–*C*). As expected (31), deletion of *Fgfr2* or *Fzd5* reduced alveolosphere growth (Fig. 2 *A*–*C*). However, sgRNAs targeting *Ctnnb1*, which encodes ß-catenin and is essential for lung development and AT2 cell proliferation (32, 33), did not. Rather than reducing alveolosphere growth, the *Ctnnb1* sgRNAs enhanced it dramatically (Fig. 2 *A*–*C* and *SI Appendix*, Fig. S4 *A* and *B*). *Ctnnb1* mutations in cancer that eliminate residues required for ß-catenin degradation promote ligand-independent Wnt signaling (34). Our *Ctnnb1* sgRNAs (hereafter referred to as *Ctnnb1^active^*) had generated alternative splice junctions that eliminated the phosphorylation sites driving ß-catenin degradation, resulting in the induction of Wnt target genes (*SI Appendix*, Fig. S4 *C* and *D*).

Overall, we identified 6 less characterized gene KOs that increased alveolosphere size and 18 gene KOs that decreased alveolosphere size and/or numbers (Fig. 2 *A*–*E*). These genes included receptors (*Erbb2, Erbb3, Tlr2),* transcription factors (*Tgif1*), kinases (*Csnk1a1, Csnk2a1*), enzymes regulating ubiquitination (*Huwe1, Bap1, Fbxw11*), and membrane-bound signaling regulators (*Tns1, Dram1*) (Fig. 2 *A*–*C*). Curiously, loss of the tumor suppressor *Keap1* decreased growth. Other genes were required for normal alveolosphere morphology (Fig. 2*E*). Alveolospheres fell into three categories having: i) typical dense morphology; ii) cystic morphology with a hollow lumen (for example, *Nkx2.1* KOs); or iii) were less spherical with budding protrusions (for example, *Elovl1*, *Csnk2a1*, *Ctnnb1,* and *Atp6v0e* mutants). The transcriptional correlates of this morphology are discussed below.

We selected *Erbb2* and *Erbb3* encoding HER2 and HER3, respectively, for further characterization. The related receptor EGFR has prominent roles in tumorigenesis and lung regeneration (35–37), whereas *Erbb2* and *Erbb3* are mutated in 1 to 2% of lung cancers and have not been associated with AT2 proliferation or stem cell activity. HER2/3 inhibition is associated with drug-induced interstitial lung fibrosis, a disease that begins with AT2 stem cell dysfunction, but this effect has been attributed to the role of HER2 in fibroblasts (38). Bulk RNA sequencing of adenocarcinomas has suggested that *Erbb2 and Erbb3* are not expressed in healthy lung tissue but can be upregulated in response to inflammatory cues during tumorigenesis (39, 40). Given that deletion of *Erbb2* or *Erbb3* in AT2 cells reduced the size and number of alveolospheres (Fig. 2*F*), we hypothesize that, contrary to previous reports, *Erbb2/3* are expressed in healthy AT2 cells and promote proliferation prior to tumor development. Single cell RNA sequencing (scRNAseq) data (17) confirmed that *Erbb2* and *Erbb3* are expressed in healthy mouse and human AT2 cells comparable to or more than *Egfr* (Fig. 2*G*). Immunostaining of alveolospheres confirmed that AT1 and AT2 cells expressed HER2 (*SI Appendix*, Fig. S4*E*). Alveolospheres treated with NRG1, the ligand for HER2 and HER3, grew better than controls (Fig. 2 *H* and *I*). Others have seen this but the effect of NRG1 was attributed to EGFR (5). Deletion of *Egfr* or *Erbb4* from AT2 cells had no discernible effect on alveolosphere growth (Fig. 2*F*) but neither gene was expressed sufficiently for us to confirm the KOs by RNA sequencing. Eliminating EGF from the alveolosphere medium prevented alveolosphere growth (*SI Appendix*, Fig. S5) but lung fibroblasts also express *EGFR* (Fig. 2*G*). Therefore, the need for EGFR in AT2 cells in this setting remains uncertain.

We interrogated the DEPMAP database (41) to determine if human cancers originating from AT2 cells require *ERBB2* or *ERBB3*. Across 93 non–small cell lung cancer lines, knockdown of *ERBB2*, *ERBB3*, or *EGFR* compromised cell line fitness, whereas *ERBB4* was dispensable (Fig. 2*J*). We conclude that HER2 and HER3 have an unrecognized role in the activity of AT2 stem cells, which may explain why therapeutic strategies that target HER2 and HER3 in lung cancer frequently cause drug-induced pulmonary fibrosis (42).

### Charting the Phenotypic Landscape of AT2 Stem Cells.

**Fig. 3.**
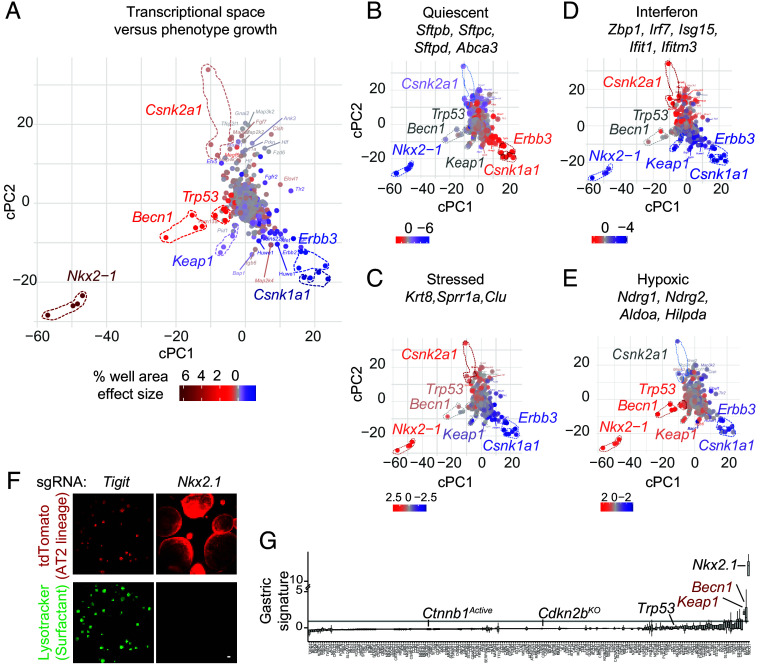
Phenome-transcriptome landscape of AT2 cell biology. (*A*–*E*) cPCA of murine alveolosphere transcriptomic profiles (based on the top 3,000 highly variable genes). Each dot represents the transcriptome from a single replicate of the indicated KO plotted in the space defined by cPC1 and cPC2. Dot color indicates total alveolosphere growth from Fig. 2*C* (*A*) or the scaled expression of the indicated gene signature (*B*–*E*). (*F*) *Tigit^KO^ and Nkx2.1^KO^* tdTomato^+^ alveolospheres stained with Lysotracker. (Scale bar, 500 μm.) (*G*) Expression of a gastric signature in alveolospheres across all gene KOs (*Materials and Methods*). New genes which significantly affect the indicated signature shown in red.

We analyzed the transcriptional changes produced by each gene KO (43) and how these correlated with imaging-based phenotypes. To survey broad features in our data and assess consistency across replicates, we first computed contrastive principal components (cPCA) for the AT2 transcriptome. This variant of PCA highlights changes relative to a control dataset, in this case *Tigit^KO^*. cPCA of the fibroblast transcriptome is discussed below. Three main axes of variability were identified (Fig. 3*A*). The first axis was enriched for increased expression of the surfactant machinery and correlated with reduced alveolosphere growth (Fig. 3 *A* and *B*), suggesting a cyclical relationship wherein AT2 cells largely produce surfactant while their stem cell activity is quiescent. The other two axes were enriched with markers of the “transitional” AT2 state that is observed after injury, including *Krt8, Sprr1a,* and *Clu* (Fig. 3*C*). It is debated whether the transitional gene signature reflects a normal intermediate state as AT2 cells differentiate into AT1 cells (25, 26) or an aberrant cell fate that impedes tissue repair (24, 26). In our dataset, the Krt8^+^ transitional AT2 state was represented by two distinct trajectories that varied from each other as much as they varied from quiescent AT2 cells (Fig. 3*C* and *SI Appendix*, Fig. S6*A*).

One trajectory featured the upregulation of interferon-stimulated genes, including *Isg15, Ifit1, Ifitm3,* and *Irf7* (Fig. 3*D*). KOs that reduced alveolosphere growth inhibited the expression of this module, indicating a connection to AT2 stem cell activity. However, eliminating other genes, including *Csnk2a1,* upregulated the interferon signaling module despite a modest growth defect. Therefore, enhanced interferon signaling does not linearly scale with AT2 cell proliferation. Cells with the interferon signature lacked a hypoxia gene signature (Fig. 3*E*), suggesting that they differed from previously defined *Krt8*^+^ subpopulations (24, 26). To determine whether the interferon response in the mutant cells was physiologically relevant, we analyzed scRNAseq data from the lungs of eight healthy donors and eight patients with interstitial lung disease (*SI Appendix*, Fig. S6 *B*–*D*). A distinct population of AT2 cells (cluster 29 in *SI Appendix*, Fig. S6*C*) exhibited upregulation of interferon-stimulated genes (*SI Appendix*, Fig. S6*D*), indicating that interferon signaling is also relevant to human AT2 cell biology.

The second trajectory with features of transitional cells expressed genes linked to hypoxia, including *Ndrg1*, *Ndrg2*, *Aldoh*, and *Hilpda* (Fig. 3*E*), and oxidative stress, including *Gsta4, Gpx2, Gpx1*, and *Gsto1* (*SI Appendix*, Fig. S6*E*)*. Nkx2.1* loss was at the pinnacle of this axis, eliciting the most alveolosphere growth and the highest number of differentially expressed genes (Fig. 3*A*). In cancer, genetic or epigenetic loss of *Nkx2.1* (44–46) drives tumors to a gastric-like state with heightened invasiveness and resistance to immunotherapy (44, 47–49). In animal studies, *Nkx2.1* deletion abrogates the lung identity of alveolar epithelial cells (50). Importantly, *Nkx2.1* deletion in AT2 cells produced a similar phenotype in alveolospheres, causing reduced lysotracker staining (Fig. 3*F*), the loss of alveolar markers, and the upregulation of gastric markers (*SI Appendix*, Fig. S6*F*).

In the absence of inactivating *Nkx2.1* mutations, the mechanisms that suppress alveolar fate and upregulate gastric fate during cancer development are unknown. Other gene KOs increased alveolosphere growth but did not upregulate the gastric program (for example *Trp53^KO^*, *Cdkn2b^KO^*, or *Ctnnb1^Active^*), suggesting no intrinsic link between proliferation and a loss of identity (Fig. 3*G*). *Keap1* and *Becn1* suppressed the gastric-like state (Fig. 3*G*), but their loss had divergent effects on alveolosphere growth. *Becn1^KO^* increased and *Keap1^KO^* decreased alveolosphere growth (Fig. 3*A*). *Keap1* and *Becn1* participate in responses to oxidative (51) and protein stress (52), respectively. Therefore, pathways mitigating cellular stress are crucial for maintaining AT2 cell identity, at least in these in vitro conditions.

### Uncovering Gene Expression Subroutines with Independent Component Analysis (ICA).

Most of our imaging-based phenotypes aligned with features from canonical PCA, but we found no transcriptional correlate for the budding morphology (Fig. 2*D* and *E*). We compared samples with budding morphologies (for example, *Elovl1*, *Csnk2a1*, *Ctnnb1^active^,* and *Atp6v0e* mutants) to controls and identified 188 differentially expressed genes (Dataset S3). Budding alveolospheres upregulated genes linked to Wnt pathway activation, including *Axin2*, *Tcf7*, *Sox9,* and *Ctnnd2,* and downregulated AT1 marker genes, including *Rtkn2, Spock, Aqp5,* and *Hopx* (*SI Appendix*, Fig. S7 *A*–*C*). This result implies there is less differentiation of AT2 cells into AT1 cells in budding alveolospheres. Our data are consistent with mouse genetic studies showing that Wnt signaling hampers AT2 cell differentiation (6). While *Csnk2a1* and *Ctnnb1* participate in Wnt signaling (53–55), it is unclear how *Elovl1* and *Atp6v0e* regulate AT2 stem cell activity.

To comprehensively identify transcriptional modules altered in a subset of KOs, we performed differential expression analysis independently for each target (Datasets S4 and S5). We then used ICA on the gene-level effect sizes to identify coregulated genes and the KOs influencing their expression (Dataset S6). This unbiased analysis identified one component (ICA17) that was enriched for Wnt target genes and activated by mutants with a budding morphology (*SI Appendix*, Fig. S7*D*). The Wnt pathway drove ICA17 gene expression because it was reduced by deletion of *Fzd5,* the Wnt ligand receptor on AT2 cells (56), or *Porcn,* the enzyme for Wnt ligand secretion (57). Beyond this component, we identified a plethora of distinct transcriptional modules that varied within a subset of KOs. Some of these modules correlated with large trends in our data such as growth (ICA8; *SI Appendix*, Fig. S7*E*) and interferon signaling (ICA13; Dataset S7). Other modules were agnostic to these large trends and altered distinct processes such as antigen presentation (ICA12) and endoplasmic reticulum stress (ICA18; Dataset S7). This analysis also revealed unexpected functional connections. For example, loss of *Slc34a2,* a transporter whose loss results in microlithiasis, activated a module enriched in the transitional *Krt8*^+^ state associated with fibrosis (ICA5; *SI Appendix*, Fig. S7*F*). How Slc34a2 regulates AT2 identity is an avenue of future study.

### AT2 Stem Cells Shape Their Niche.

**Fig. 4.**
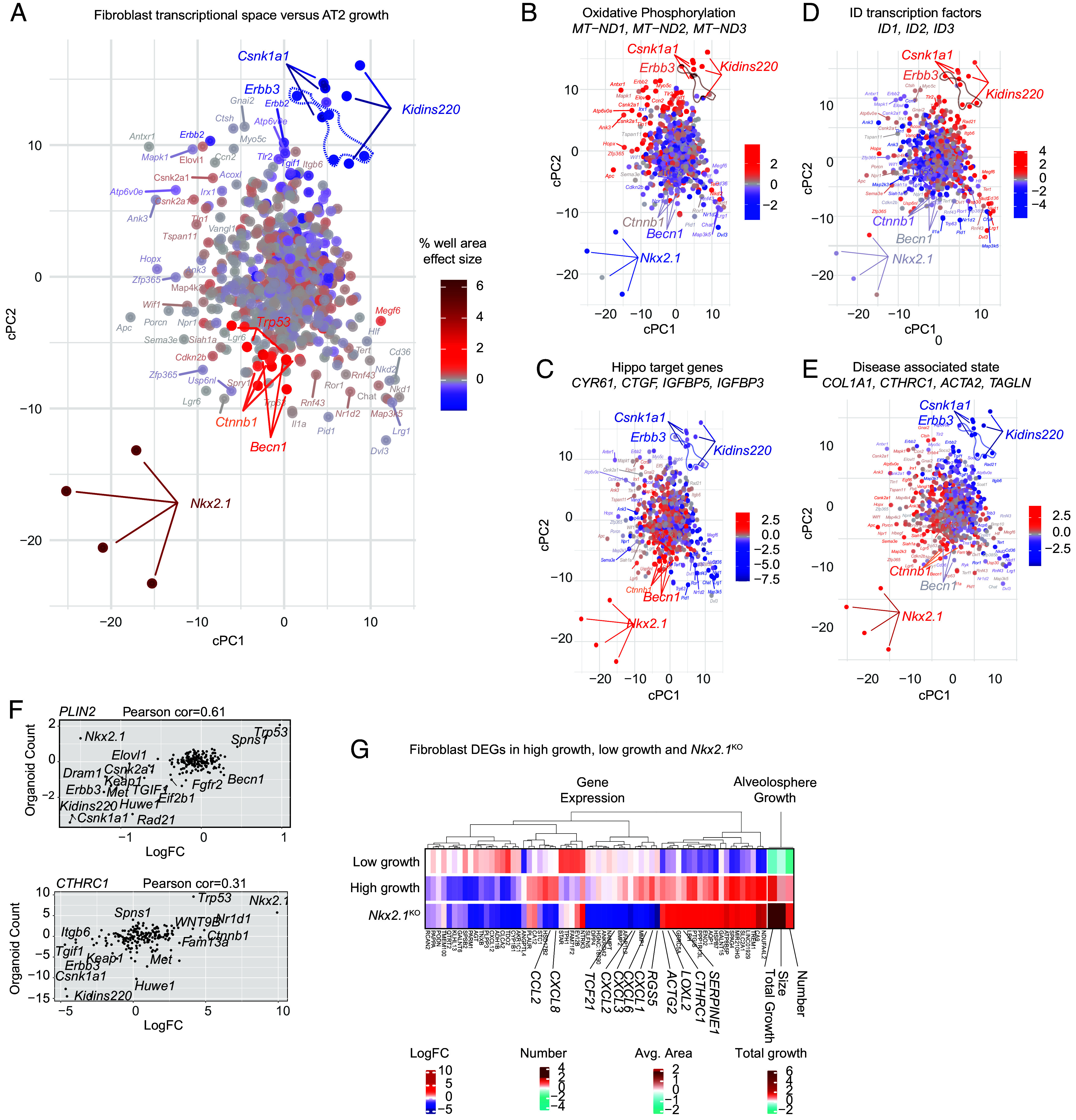
AT2 cells shape their fibroblast niche. (*A*–*E*) cPCA of the transcriptomes of human fibroblasts in chimeric cocultures (based on the top 3,000 highly variable genes). Dots represent individual replicates of the indicated KOs plotted in the space defined by cPC1 and cPC2. Dot color shows total alveolosphere growth from Fig. 2*C* (*A*) or expression of the indicated gene signature (*B*–*E*). (*F*) Scatter plot showing the relationship between fibroblast *PLIN2* (*Upper*) and *CTHRC1* (*Lower*) differential expression [Log fold change (LogFC), x-axis] and alveolosphere growth (effect size; *Materials and Methods*, y-axis) across different AT2 gene KOs. Dots represent the median level across replicates of each KO. (*G*) Heatmap showing the top differentially expressed genes between fibroblasts in low (*Top*) vs. high growth (*Middle*) wells as measured by imaging (green-to-brown heatmap, *Right*), and how their expression changes in *Nkx2.1^KO^*wells (*Bottom*).

We analyzed the non-cell-autonomous effect of each gene KO on neighboring fibroblasts through cPCA analysis. Fibroblast transcriptomes formed three major groups, but replicates from different KOs were intermixed within these broad clusters, suggesting fibroblast transcriptomes are more homogenous than those of AT2 cells (Fig. 4*A*). This result may be expected because: i) changes to the fibroblast transcriptome were a secondary, non-cell-autonomous effect, and ii) fibroblasts yielded fewer reads than AT2 cells in sequencing (Fig. 1*C*). Nonetheless, gene KOs in AT2 cells influenced a diverse set of fibroblast processes, including metabolism (Fig. 4*B* and *SI Appendix*, Fig. S8*A*), Hippo signaling (Fig. 4*C*), and ID transcription factors (Fig. 4*D*).

We also observed a signature of the *Cthrc1^+^* population that is found in various injury and cancer settings (Fig. 4*E*; *CTHRC1, ACTA2, TAGLN*) and proposed to be a major driver of fibrotic remodeling (58, 59). Signals from infiltrating macrophages and monocytes are hypothesized to promote differentiation of fibroblasts to the *Cthrc1^+^* state (60–64). Our results suggest that local paracrine signals from proliferating AT2 cells may contribute to this fibroblast heterogeneity.

**Fig. 5.**
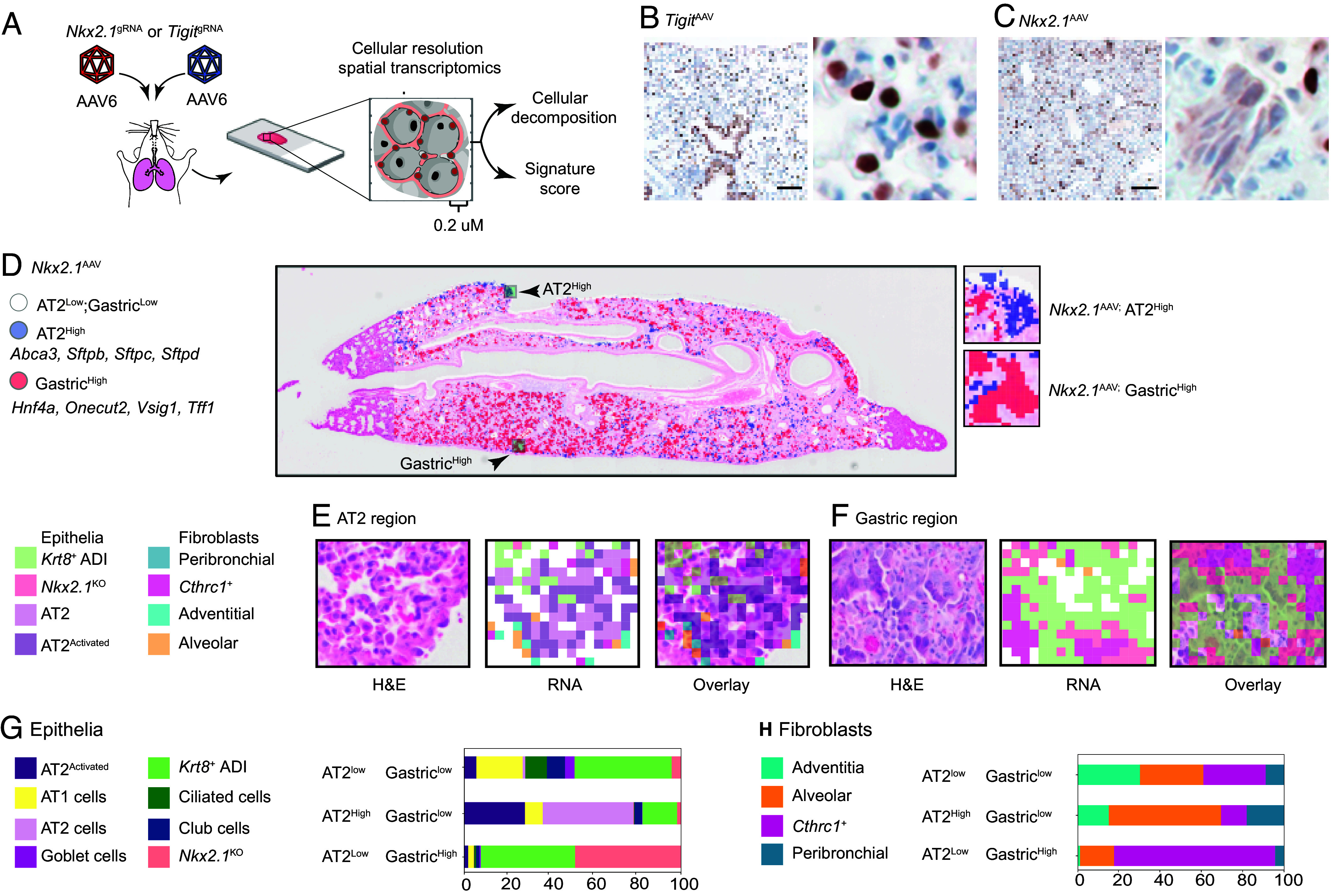
Spatial transcriptomics charts the effect of *Nkx2.1^KO^* on surrounding cells. (*A*) AAV-mediated KO of *Nkx2.1* in mice. (*B* and *C*) Immunolabeling of Nkx2.1 (brown) in mouse lung sections. (Scale bar, 100 µm.) (*D*) Hematoxylin and eosin–stained (H&E) lung section overlaid with signature plots for AT2 cells (blue) and gastric cells (red). Insets (*Right*) provide closeup representative images of regions enriched for signatures of AT2 (*Top*) and gastric (*Bottom*) cells. (*E* and *F*) Lung sections stained with H&E (*Left*), colored for cellular composition (RNA, *Center*), and an overlay of the two (*Right*). (*G* and *H*) Composition plots show the relative distribution of cell populations within regions lacking either signature (*Top*), enriched in the AT2 signature (*Middle*), or enriched in the gastric signature (*Bottom*) across epithelial (*G*) and stromal (*H*) compartments. n = 2 animals per group.

Changes in the fibroblast transcriptome correlated with alveolosphere growth. Expression of Hippo pathway genes, including *CTGF*, *CYR61*, and *IGFBP7* (Fig. 4 *A* and *C*), glycolytic components (*SI Appendix*, Fig. S8*A*), and the *Cthrc1* signature all correlated with alveolosphere growth (Fig. 4 *A* and *E*). Conversely, reduced alveolosphere growth was linked to increased expression of the transcription factor genes *ID1, ID2,* and *ID3*, as well as genes regulating oxidative phosphorylation (Fig. 5*B* and *SI Appendix*, Fig. S8*B*), including *MT-ND1, MT-ND2,* and *MT-ND3*. These findings suggest that AT2 cells coordinate tissue responses by connecting epithelial regeneration to fibroblast-mediated healing and metabolic reprogramming.

AT2 cell proliferation and identity were the two most influential factors on the fibroblast transcriptome (Fig. 4*A*). This observation prompted us to investigate how the coordination between alveolosphere growth and fibroblast transcription was altered by the loss of AT2 cell identity. First, we identified the most impacted fibroblast genes as a function of alveolosphere growth by performing differential expression between KOs that exhibited “high alveolosphere growth” vs. “low alveolosphere growth.” We then determined how their expression was affected by *Nkx2.1^KO^*, which elicits robust growth but a loss of AT2 cell identity. Expression of *PLIN2*, a marker of “lipo-fibroblast” transcriptional modules considered specific to alveolar fibroblasts and thought to support surfactant production (65, 66), scaled with increased AT2 growth but was abrogated by *Nkx2.1^KO^* (Fig. 4*F*). Other markers of lipo-fibroblasts were also downregulated by *Nkx2.1^KO^* (*SI Appendix*, Fig. S8*C*), indicating that alveolar-specific fibroblast gene expression depends on interactions with AT2 cells. Expression of fibroblast-derived chemokine transcripts also correlated with alveolosphere growth but was abrogated by *Nkx2.1^KO^* (Fig. 4*G*). Thus, AT2 stem cell activity appears to influence immune cell recruitment by fibroblasts. This finding may explain why *NKX2.1* deficiency in lung cancer is prognostic of a muted response to immunotherapy. More broadly, our data reveal that these alveolar-specific fibroblast programs represent a cellular state dynamically regulated by paracrine signaling rather than a cell-intrinsic lineage program. Hence, *Nkx2.1* is not just a master regulator of lung epithelial identity (67), it also orchestrates lung-specific fibroblast transcriptional programs.

Other fibroblast modules scaled with alveolosphere growth even when AT2 cell identity was lost. Markers of disease-associated fibroblasts and “wound-healing,” including *CTHRC1*, *ACTA2*, *TAGLN*, and *SERPINE1*, behaved in this manner (Fig. 4 *F* and *G*). We propose that these modules represent more generalized coordination between epithelia and fibroblasts across different organs. Consistent with this idea, *CTHRC1* is upregulated in fibroblasts across a variety of organs following epithelial injury (59). Differential expression and gene set enrichment analysis charts which of these coregulated programs are likely to be lung-specific (i.e., expression of certain cytokines in response to epithelial repair) and which are agnostic to AT2 cell identity (i.e., upregulation of fibrotic and glycolytic machinery; Fig. 4*G* and *SI Appendix*, Fig. S8*D*).

### *Nkx2.1* in Epithelial Cells Impacts Fibroblast Subpopulations in the Mouse Lung.

To confirm that *Nkx2.1* governs fibroblast behavior in vivo, we infected Cas9-expressing mice with adeno-associated virus 6 (AAV6) expressing *Nkx2.1*-targeting gRNAs (*Nkx2.1*^AAV^; Fig. 5*A*). Control mice were infected with AAV6 expressing *Tigit-*targeting gRNAs (*Tigit*^AAV^). The viruses were delivered via intratracheal injection to selectively target the lung epithelium (68). The lungs of *Nkx2.1*^AAV^ mice had high numbers of alveolar macrophages and neutrophils in the alveoli and alveolar septa, while lymphocytes and plasma cells cuffed blood vessels and airways in both groups of mice (*SI Appendix*, Fig. S9 *A* and *B*). The perivascular and peribronchiolar lymphoplasmacytic infiltrates across both animal groups indicate that both *Nkx2.1*^AAV^ and *Tigit*^AAV^ initiated an injury response.

Consistent with previous reports (44, 69), deletion of *Nkx2.1* elicited adverse effects, including weight loss. The alveolar parenchyma of animals receiving *Nkx2.1*^AAV^ contained individual or tuft-like aggregates of pleomorphic transformed cells that extended into the alveolar spaces (*SI Appendix*, Fig. S9*B*). These cells had enlarged nuclei and cytoplasm (karyomegaly and cytomegaly) compared to AT2 cells, and increased cytoplasmic-to-nuclear ratios, which is consistent with previous analyses of *Nkx2.1* deficiency in vivo (44, 69). Immunolabeling of Nkx2.1 yielded a clear nuclear signal in the airway and alveoli of mice infected with *Tigit*^AAV^ (Fig. 5*B*). Lungs infected with *Nkx2.1*^AAV^ exhibited weak labeling in patches, indicating a mosaic pattern of *Nkx2.1* deletion (Fig. 5*C*). Nuclear Nkx2.1 was noticeably diminished in the foci of transformed, tuft-like cells.

To determine how *Nkx2.1* deletion in AT2 cells affected the surrounding fibroblasts, we performed spatial transcriptomics at cellular resolution in lungs infected with *Nkx2.1*^AAV^ using VisiumHD (70). We mapped this spatial data onto a reference derived from multiple lung injury datasets (*SI Appendix*, Fig. S9 *C* and *D*) (25, 71, 72) using robust cell type decomposition (RCTD) (73) to identify individual cell types within each spot on the tissue section. We captured all major populations in our reference atlas, including normal AT2 cells, activated AT2 cells, and *Krt8*^+^ cells (Fig. 5 *E*–*G*). As expected, we also observed an epithelial population expressing a gastric signature (Fig. 5*E* and *SI Appendix*, Fig. S9*E*; *Onecut2, Hnf4a, Vsig1, Tff1*).

We divided the *Nkx2.1*^AAV^ lung sections into regions using a 50 um^2^ grid (Fig. 5 *D*–*F*). 24,351 regions were enriched in the gastric signature, presumably driven by *Nkx2.1* deletion. 11,311 regions were enriched in AT2 cells and served as an in situ reference for the effect of gastric differentiation. As expected, *Nkx2.1^KO^* cells localized almost exclusively to the gastric regions (Fig. 5 *E* and *G*). Regions enriched in the AT2 signature contained equal parts homeostatic AT2 cells and those participating in an injury response (Fig. 5 *E* and *F*; *Krt8*^+^ alveolar differentiation intermediate (ADI) or AT2^Activated^), indicating an ongoing repair process in these areas. We compared the distribution of fibroblast subpopulations within each region (Fig. 5*H*). Consistent with their role as AT2 niche cells, alveolar fibroblasts were the most well-represented stromal population within AT2^high^ regions, whereas *Cthrc1*^+^ fibroblasts were relatively rare (Fig. 5 *E* and *H*). Notably, there were 70% fewer alveolar fibroblasts in gastric^high^ regions, while *Cthrc1*^+^ fibroblasts increased approximately 650%. Gastric^high^ regions were also depleted of adventitial and peribronchial fibroblasts (Fig. 5 *E* and *H*). The distinct fibroblast subpopulations within gastric^high^ vs. AT2^high^ regions of the lung indicate that the non-cell-autonomous phenotype was highly localized. Regions that lacked expression of both gastric and AT2 signatures were less enriched for a particular fibroblast subpopulation (Fig. 5*H*). Together, these data suggest that *Cthrc1*^+^ fibroblast numbers grow in response to signals from *Nkx2.1*-deficient cells, rather than from the absence of signaling by AT2 cells. Consistent with our alveolosphere data, fibroblasts in gastric^high^ regions expressed fewer chemokine transcripts (*SI Appendix*, Fig. S9*F*), which predicts changes to the immune microenvironment.

To test this hypothesis, we analyzed immune cells close to cells that had lost *Nkx2.1* (as defined by the loss of AT2 markers and gain of gastric markers) and compared them to those next to “activated” AT2 cells, which is the transcriptional state activated when AT2 cells are in an inflammatory setting (25, 74). The immune microenvironment of gastric cells was enriched for alveolar macrophages and T cells but depleted of infiltrating neutrophils and non-classical monocytes (*SI Appendix*, Fig. S9*G*). Thus, Nkx2.1 influences chemokine expression in fibroblasts and the immune microenvironment in the adult lung as well as in cultured alveolospheres.

To separate the effects of *Nkx2.1* deficiency from the immune response to AAV infection, we analyzed scRNAseq data from *Tfc2pl1^CreERT2.LSL.EYFP^ Nkx2.1^fl/fl^* mouse lungs (72), where *Nkx2.1* was selectively deleted in epithelial cells after tamoxifen treatment (*SI Appendix*, Fig. S10*A*). Within the epithelial populations, *Nkx2.1* deletion reduced expression of the AT2 markers *Sftpc*, *Cldn18*, and *Slc34a2*, while upregulating the gastric markers *Hnf4a* and *Onecut2* (*SI Appendix*, Fig. S10 *B*–*G*). *Col1a1^+^* fibroblasts were identified in control and *Nkx2.1*-deficient lungs (*SI Appendix*, Fig. S10*H*). Differential expression analysis revealed that fibroblasts in *Nkx2.1-deficient* lungs upregulated genes for extracellular matrix production, cell proliferation, and glycolysis, as well as the signature for *Cthrc1*^+^ fibroblasts, whereas markers of homeostatic lung fibroblasts and ID transcription factor genes were expressed less (*SI Appendix*, Fig. S10*I*). These data provide further support for the notion that AT2 identity impacts transcriptional programs in lung fibroblasts.

## Discussion

The primary advantage and limitation of our alveolosphere system is the chimeric nature of the organoids. Cell-type specific information is obtained without dissociating or sorting the two cell types, greatly enhancing throughput. However, not all signaling interactions are conserved between murine and human cells, presenting the possibility of false negatives. In addition, coverage from the transcriptomics was insufficient in most samples to directly assess the nature of the CRISPR-mediated gene edits, raising the possibility that gain-of-function mutations besides those in *Ctnnb1* were overlooked. Immune and endothelial populations, which may contribute to AT2 function in concert with fibroblasts, are also lacking from this reductionist organoid model. Therefore, many of the hypotheses generated by this multilayered dataset will require further validation in future studies with more complex animal models or precision cut lung slices.

The chimeric screening platform complements similar Cas9-based screening approaches in fetal lung epithelial progenitors (75) but may better interrogate disease-relevant biology by using physiologically mature AT2 cells. Moreover, by capturing imaging and transcriptomic information for each well, we could interpret changes to organoid morphology that are typically ignored. We found that morphological changes were not linked to growth. For example, KO of *Nkx2.1* and *Csnk1a1* produced cystic structures but had opposing effects on organoid size. *Erbb3* and *Becn1* KOs both produced dense organoids but were distinguished by the size of their organoids. Thus, organoid morphology probably reflects a cellular process unique from growth.

The chimeric system revealed that AT2 cells can choreograph an injury response by affecting intrinsic fibroblast behaviors like extracellular matrix production and metabolism, and by influencing how fibroblasts signal to other populations such as immune cells. A large catalog of AT2 cellular phenotypes shaped fibroblast transcriptional programs. Some AT2 cell-to-fibroblast signaling is likely lung-specific because many fibroblast features were lost when AT2 cell identity was abrogated by *Nkx2.1* deficiency. In developmental settings, such as feather or hair formation, mesenchymal cells like fibroblasts specify the organ identity of epithelial cells (76). Our data invert this paradigm because lung-specific fibroblast programs relied on juxtacrine signaling from AT2 cells.

How the AT2 stem cell shapes the niche is intrinsic to its identity. Loss of AT2 cell identity following *Nkx2.1* deletion profoundly reshaped the fibroblast transcriptome. This observation is relevant to lung adenocarcinoma because loss of *NKX2.1* and/or AT2 cell identity produces aggressive tumors that are refractory to immunotherapy (49, 77). The effect of *Nkx2.1* loss on fibroblasts suggests that lineage-switching may suppress the ability of other cells to activate the immune response. Future studies exploring the pathways underlying this effect may identify new therapeutic targets in lung disease.

Our screen revealed a previously unappreciated role for *Erbb2/3* in stimulating AT2 stem cell activity. This result sheds light on clinical trial outcomes for *ERBB2*-mutant lung cancers treated with trastuzumab-deruxtecan, a ERBB2/HER2-neutralizing antibody conjugated to a topoisomerase inhibitor. Perplexingly, patients lacking an activating *ERBB2* mutation also responded to the drug (42). In addition, 12% of patients in this trial developed interstitial lung disease. We show that normal AT2 cells express *Erbb2/3* and respond to the ligand NRG-1, so most AT2 cell-derived lung adenocarcinomas may express HER2/3 irrespective of their *ERBB2* mutational status. Given that AT2 cell dysfunction is known to cause interstitial lung disease and fibrosis (78, 79), the adverse effects seen in the clinic could indicate on-target effects on healthy AT2 cells. Of note, expression of *Erbb2/3* RNA and HER2 protein is detected in both AT2 and AT1 cells. Our data show these receptors are necessary for AT2 proliferation, although we have not identified a role for Her2/3 signaling in AT1 cells. Future studies will explore whether these receptors also play a role in AT1 differentiation or function.

Inhibition of AT2 stem cell activity by a variety of gene KOs led to higher surfactant expression, perhaps indicating that AT2 cells toggle between their secretory and stem cell functions. It will be interesting to explore how this dynamic affects lung injury, since both processes are important for gas exchange.

## Materials and Methods

All mouse studies complied with relevant ethics regulations and were approved by the Genentech Institutional Animal Care and Use Committee in an Association for Assessment and Accreditation of Laboratory Animal Care (AAALAC)-accredited facility in accordance with the Guide for the Care and Use of Laboratory Animals and applicable laws and regulations. *Sftpc-CreERT2 Rosa26.LSL.tdTomato* mice (4) were dosed with 10 mg/kg tamoxifen in sunflower seed oil by intraperitoneal injection on three consecutive days. tdTomato^+^ AT2 cells were isolated 3 wk later.

Detailed methods and reagents are provided in *SI Appendix*, *Materials and Methods*.

## Supplementary Material

Appendix 01 (PDF)

Dataset S01 (XLSX)

Dataset S02 (XLSX)

Dataset S03 (XLSX)

Dataset S04 (XLSX)

Dataset S05 (XLSX)

Dataset S06 (CSV)

Dataset S07 (XLSX)

## Data Availability

RNAseq and Visium HD data have been deposited in GEO (Accession: GSE307351) (43). All other data are included in the manuscript and/or supporting information.
