# Supplementary material for: Mapping the dialogue: Decoding alveolar stem–niche interactions
Source: Proc Natl Acad Sci U S A. 2026 Jul 8;123(28):e2606113123. doi: 10.1073/pnas.2606113123 (PMC13367804; doi:10.1073/pnas.2606113123)
Supplement: Supplementary file 1 — Appendix 01 (PDF) [file pnas.2606113123.sapp.pdf]

PNAS

Supporting Information for
Mapping the Dialogue: Decoding Alveolar Stem-Niche Interactions.

Ahmad N. Nabhan, Anne Biton, Christine Everett, Conrad Foo, Diana Wu, Joshua D. Webster, Alina A. Alam, Elisa Penna, Sandra Rost, Neha Rohatgi, Rohit Reja, Ranel J. Tulpano, Shiqi Xie, Celine Eidenschenk, Kim Newton, Joseph R. Arron, Vishva M. Dixit

Corresponding authors: Vishva M. Dixit, Ahmad N. Nabhan
Email: dixit@gene.com, nabhan@berkeley.edu

This PDF file includes:

Supplementary Text
Figures S1 to S10

Other supporting materials for this manuscript include the following:

Datasets S1 to S7

Supplementary Information Text

Materials and Methods

Mice
Infections with AAV6 viruses (Virovek) expressing *Nkx2.1* (GACGGCGCCGTGGTGCCCCA and CGCAGCGTACAGACAGGGCC) or *Tigit* (GCTGACATACATACAGGCCT and CATGCATGGCTGGCTGCTCC) gRNAs were performed by the Jackson Laboratory (Sacramento, CA, USA). Female and male *Rosa26*-Cas9 knock-in mice (strain 026179) aged 8-12 weeks received 10^{12} viral genomes by intratracheal delivery on days 0 and 7. Lungs were harvested for analyses on days 13-20 ($Nkx2.1^{AAV}$) or day 22 ($Tigit^{AAV}$).

Cell Isolation and Sorting
Mouse lung cells were isolated as described (1) with minor modifications. Briefly, lungs were inflated with a protease solution cocktail (5 U/mL Dispase, 450 U/mL Collagenase Type I, 4 U/mL Elastase, and 0.33 U/mL DNase I in DMEM/F12), removed en bloc, minced, and digested for 30 min at 37°C with shaking at 100 rpm. Proteases were inactivated by the addition of 10% fetal bovine serum and the cells passed through a 100 µm filter. Red blood cells were removed using ACK lysis buffer, and the remaining cells passed through a 70 µm filter. $tdTomato^{+}$ AT2 cells were sorted in a FACS Aria (BD Biosciences). Human fibroblasts were sorted as a $Epcam^{neg}$, $CD31^{neg}$, $CD45^{neg}$ populations from de-identified human samples, expanded for two passages and cryopreserved. For each alveolosphere experiment, cells from the same patient were thawed and used at this same passage for each experiment (P3).

RNP Complex Formation and Electroporation
Ribonucleoprotein (RNP) complexes were formed by mixing 6 µL of 8.3 µM Cas9 (IDT) with 3 µL of 100 µM gRNA (Synthego) for up to 2 h. After freshly sorted mouse AT2 cells were resuspended at 4 x 10^5 cells/mL in P3 buffer, 15 µL of cells and 1 µL of 100 µM electroporation enhancer (IDT) were added to the RNP complexes. The cells were electroporated in 96-well format (using only the inner 60 wells) with a 4D nucleofector (Lonza; pulse code CM-113) and placed on ice.

Cell Co-culture and Transwell Seeding
Immediately after AT2 cell nucleofection, we added 100 µL of chilled normal human lung fibroblasts at 6 x 10^5 cells/mL in DMEM, 10% FBS, 1x Penicillin-Streptomycin to the AT2 cells. The cell mixtures were incubated at 4°C for 30 min. Meanwhile, Matrigel plates were prepared by adding 120 µL of growth factor reduced Matrigel (Corning) to the 60 inner wells of a 96-well plate and kept at 4°C. Finally, all cells from the nucleofection plate were transferred to the pre-chilled Matrigel plate and mixed thoroughly. 40 µL of cell-Matrigel mix was transferred to the apical chamber of four replicate 96-well Transwells (Corning). Edge wells were pre-filled with PBS to prevent plate-edge effects. Plates were placed at 37°C to solidify the Matrigel, and then 150 µL of MTEC/Plus medium was placed in the basal chamber. The basal media was refreshed 3 times per week for 2 weeks. Plates were imaged using ImageXpress (Molecular Devices) on days 7 and 14. After imaging on day 14, RNA was extracted using a RNeasy 96 kit (Qiagen).

Image Processing
Segment Anything Model (SAM) v1 from Meta (2) was used to segment the organoids within each well image. For compatibility with SAM, 16-bit images were transformed into 8-bit format, ensuring each pixel was encoded within a range of 0 to 255. This conversion was achieved using OpenCV's (3) convertScaleAbs function, followed by conversion to RGB format using the Python Imaging Library (4). The SAM model was initialized using a pre-trained checkpoint (sam_vit_h_4b8939.pth) and deployed on a GPU for efficient computation. The SamAutomaticMaskGenerator function (parameters crop_n_layers=1, crop_n_points_downscale_factor=2) was applied to each image to generate segmentation masks. Generated masks were filtered based on area to remove overly small (<= 200) or excessively large masks segmenting the whole well area. Additional shadow filtering was applied

when < 150 segmented regions were detected, eliminating masks that completely encompassed others to reduce over-segmentation artifacts. Masks included in the margins of the well images at day 14 or in the corners of the well images at day 7 were also excluded before statistical analyses. After filtering, the number of organoids and their average size were determined for each well. To estimate the fraction of well area covered by organoids, we calculated the area of the union of bounding boxes for each image and divided it by the total image area, excluding appropriate margins or corner regions.

Embedding Extraction for Day 14 Images

The bounding box was used to extract day 14 image regions, which was then fed into the SamPredictor function to generate image embeddings. The generated embeddings were concatenated along the first axis and subjected to adaptive average pooling to reduce the spatial dimensions from 256x64x64 to 256x8x8. The resulting tensor was flattened, converting it into a one-dimensional array for each segmented organoid region. Embeddings were centered by plate, and TdTomato wells were discarded before computing cPCA (5).

Statistical Analysis of Organoid Features

Linear models were employed to compare imaging readouts (organoid count, average size, and fraction of well area covered by organoids) with *Tigit* gRNA reference wells. The data were first centered and scaled by plate to facilitate the comparison of effect sizes between days 7 and 14. Subsequently, a linear model was fitted individually to each target, using the *Tigit* gRNA wells from the same plates as reference. P-values were adjusted across targets using the Benjamini-Hochberg false discovery rate (FDR) method.

SmartSeq Library Preparation and Sequencing

Bulk RNA seq libraries were prepared from extracted RNA using a modified Smart-seq3 protocol (6). An annealing reaction was performed with purified RNA in 5% PEG 8000, 0.5 U/µL RNAse Inhibitor (Sequrna), 0.6 µM Smart-seq3 oligo dT (/5Biosg/ACGAGCATCAGCAGCATACGATTTTTTTTTTTTTTTTTTTTTTTTTTTTTTVN), 1.2 mM dNTPs at 70°C for 10 min. Reverse transcription was performed in 24 mM Tris-HCl, pH8.0, 29 mM NaCl, 2.4 mM $MgCl_2$, 97 µM GTP, 8 mM DTT, 2 µM Templet Switch Oligo (TSO; /5Biosg/AGAGACAGATTGCGCAATGNNNNNNNNrGrGrG) with 2 U/µL Maxima H Minus Reverse Transcriptase (Thermo Fisher) at 42°C for 90 min and repeated the following steps for 10 cycles: 50°C for 2 min and 42˚C for 2 min. The reaction was terminated at 85°C for 5 min.

cDNA amplification was with 0.5 µM cDNA forward (TCGTCGGCAGCGTCAGATGTGTATAAGAGACAGATTGCGCAA*T*G) and 0.1 µM cDNA (ACGAGCATCAGCAGCATAC*G*A) reverse in 2.9 mM $MgCl_2$ and 1x Kapa HiFi Hotstart Readymix (Roche). Reactions were incubated at 95°C for 3 min then subjected to 14 cycles of 98°C for 20 s, 65°C for 30 s, and 72°C for 4 min. cDNA was diluted 1:10 in water and tagmentation performed in 1x TAPS $MgCl_2$ buffer with 0.08 µL in-house purified and assembled Tn5 enzyme at 55°C for 10 min and the following adapter oligos (Tn5ME_rev: /5phos/CTGTCTCTTATACACATCT;Tn5ME_A:TCGTCGGCAGCGTCAGATGTGTATAAGAGACAG;Tn5ME_B: GTCTCGTGGGCTCGGAGATGTGTATAAGAGACAG). The reaction was quenched by addition of 0.02% SDS. Indexing was performed in Phusion HF PCR mix (NEB) with 0.1 µM of each Nextera indexing primer (Nextera_i7: CAAGCAGAAGACGGCATACGAGATXXXXXXXXGTCTCGTGGGCTCGG; Nextera_i5: AATGATACGGCGACCACCGAGATCTACACXXXXXXXXTCGTCGGCAGCGTCa). We designed 384 different Nextera i7 and i5 primer pairs to make sure every sample can be indexed separately in a 384-well format. The PCR reaction was incubated at 72°C for 5 min, 95°C for 3 min, and then had 10 cycles of 98°C for 20 s, 65°C for 30 s, and 72°C for 30 s, followed by 72°C for 4 min.

After PCR, 5 µL of samples were aliquoted from each well and pooled together. The final libraries were purified by the 0.8X AmpureXP beads (Beckman Coulter) and quantified by using Qubit dsDNA HS assay (Thermo Fisher) and TapeStation D1000 kit (Agilent) before sequencing. All

sequencing was done on Illumina NextSeq 2000 or NovaSeq X with the aim of getting 3M raw reads per sample using single-end 100 bp sequencing.

Transcriptomics

Sequencing reads were divided between mouse and human using Xenome (7). Only reads unambiguously assigned as mouse or human were aligned to their reference genome (mouse: GRCm38.p5, human: GRCh38.p10) through the HTSeqGenie v4.35 Bioconductor package (8). Reads with low nucleotide qualities (70% of bases with quality <23) or matches to rRNA and adapter sequences were removed. Remaining reads were aligned using GSNAP (9, 10) version 2013-10-10-v2 allowing a maximum of two mismatches per 75 base sequence (parameters: '-M 2 -n 10 -B 2 -i 1 -N 1 -w 200000 -E 1 --pairmax-rna=200000 --clip-overlap').

Transcript annotation was based on the Gencode genes database (human: GENCODE 27, mouse: GENCODE M15). Reads unambiguously mapping to exons were counted to get gene-level read counts. Only mouse samples with at least 7,500 detected genes and human samples with at least 10,000 detected genes were kept for subsequent analysis. Samples were normalized for library sizes using Trimmed Mean of the M-values (11). Before exploratory analysis using cPCA, gene count-per-million were corrected for plate effects with limma::removeBatchEffect function. cPCA was performed using the R package scPCA v1.19.0 (12) on the 3,000 most variable genes selected using scran v1.33.2 (13) with functions modelGeneVar, combineVar, and getTopHVGs.

Differential expression analyses were performed with limma (14) + voom (15) for each CRISPR target independently. *Tigit* and *TdTomato* gRNA wells on the same plate were used as references, and plates as covariate in the model. Given the low sequencing depth inherent to the SmartSeq technology, only genes with average expression > 1.5 in the limma+voom results were used for further analysis. Gene set enrichment analyses were performed with cameraPR (16) using the t-statistic as input value.

Independent Component Analysis

Independent Component Analysis (ICA) was used to find axes of co-regulated genes across targets. ICA was applied to the limma-voom t-statistics transformed into z-scores using the limma::zscoreT function. We used the Joint Approximation Diagonalization of Eigen-matrices (JADE) algorithm (17) implemented in the ica R package v1.0.3 (18).

Histology and TTF1/Nkx2.1 Immunohistochemistry

Formalin-fixed lungs were routinely processed, embedded, and stained with hematoxylin and eosin for histologic evaluation. Nkx2.1 immunohistochemistry was performed with 1 μg/mL mouse anti-TTF1 antibody (8G7G3/1, Thermo Scientific). Antigen retrieval was performed in a PT Module (Thermo Scientific) with Target antigen retrieval solution (Agilent). Non-specific labeling was blocked with hydrogen peroxide, ScyTek biotin block, and bovine serum albumin. The immunolabeling was detected with rabbit anti-mouse antibody (M204-3, Abcam) and a PowerVision HRP anti-rabbit detection system (Leica) with diaminobenzidine as the chromogen and hematoxylin counterstaining. Mouse lung was used as a positive control tissue, and the primary antibody was replaced with a naïve mouse IgG as a negative control.

Visium HD

Formalin-fixed and paraffin-embedded (FFPE) tissues were mounted on a cryostat O.C.T. chuck and sectioned at a thickness of 5 μm onto 6.5 mm^2 Visium HD Slides (10x Genomics). Subsequent deparaffinization, hematoxylin and eosin staining, imaging, and decrosslinking were conducted in accordance with the Visium HD FFPE Tissue Preparation Handbook. Brightfield histological imaging was performed using a 20x objective lens on an Olympus VS200 slide scanner, ensuring high-quality visualization of tissue morphology. Following imaging, tissue samples underwent further processing and sequencing steps as outlined in the Visium HD Spatial Gene Expression Reagent Kits User Guide, which includes probe release and capture facilitated by CytAssist. All steps were performed according to the recommended protocol. Libraries were

prepared according to the Visium HD Spatial Gene Expression sequencing metrics. Sequencing was performed on an Illumina NovaSeq1 system equipped with NovaSeq6000 flow cells (Illumina), utilizing a NovaSeq S2 Reagent Kit (100 cycles, Illumina). Each sample was sequenced to a depth of one billion paired-end (PE) reads, using the following run parameters: Read 1, 43 cycles; i7 index, 10 cycles; i5 index, 10 cycles; and Read2, 50 cycles.

Spatial Transcriptomics Analysis Preprocessing
The SpaceRanger pipeline v3.0 (10x Genomics) was used to convert FASTQ files from the prepared Visium HD libraries into count matrices. The 10x provided reference genome (mm10) and probe set (mouse v2) were used to decode the sequencing libraries. The CytAssist images were aligned to high-resolution H&E images using the manual Visium alignment tool after spaceranger count was run.

Construction of a Reference Single-cell Dataset
Three datasets were merged to create the single-cell reference for phenotyping: an annotated atlas consisting of bleomycin-treated mouse lungs (19), an annotated dataset enriching for fibroblast populations from bleomycin-treated lungs in transgenic mice (20), and a dataset of *Nkx2.1*-deficient epithelial cells from mouse lungs (21). The first and second datasets were annotated previously. Only cells enriched for the gastric signature were selected from the 3rd dataset. To generate a harmonized set of cell phenotypes, we co-embedded all 3 datasets using scVI (22), then used a random forest classifier to transfer fibroblast phenotypes from dataset 2 to dataset 1, and vice versa for all other phenotypes. All cells filtered from dataset 3 were labelled as *Nkx2.1* KO cells, and no labels were transferred to them. RCTD (23) was used to transfer phenotypic content from the reference single-cell RNAseq dataset to Visium HD data (binned at 8 microns). We ran RCTD in the doublet mode, using the default hyperparameters for gene selection, with a minimum cutoff of 75 transcripts per spot. Both the multiplet and doublet weights were saved.

Spatial composition of gastric and AT2 niches
Phenotypic weights for the AT2 and gastric phenotypes were used to classify pixels as enriched for these phenotypes or not. A weight greater than 20% was considered positive (determined empirically by examination). Phenotype proportions in pixels were aggregated using the doublet weight per pixel. Significant differences in proportions between enriched and non-enriched pixels were determined by fitting a logistic regression model to predict the enrichment status of each pixel based on the phenotype. 95% confidence intervals were calculated for the log-odds of each phenotype, and a phenotype was considered significantly different if the 95% confidence interval did not contain 0.

Spatial Colocalization of Cellular Phenotypes
As pixels are not cells, we did not use a label permutation test. Instead, we constructed the non-spatial null distribution by assuming that the 2-ring neighborhood of each pixel (including itself) was drawn from a Dirichlet random variable. The parameters of the Dirichlet distribution were estimated using the method of moments across all pixel neighborhoods in a sample. The marginal distribution of the expected fractional number of a given phenotype in a neighborhood was then beta distributed. Pixel neighborhoods with less than a 5% probability of being drawn from this null distribution were considered significantly interacting/non-interacting (depending on the tail) with that phenotype. Phenotype-phenotype interactions were then inferred by fitting a logistic regression model to predict interaction status based on phenotype proportions within the pixel. For analyzing immune populations neighboring *Nkx2.1* KO cells and activated AT2 cells, we focus on pixels where either cells type is observed to be the dominant transcriptome and analyzed what immune populations are listed as the “second cell” to define the neighbors. These were then tabulated as a percentage of the total.

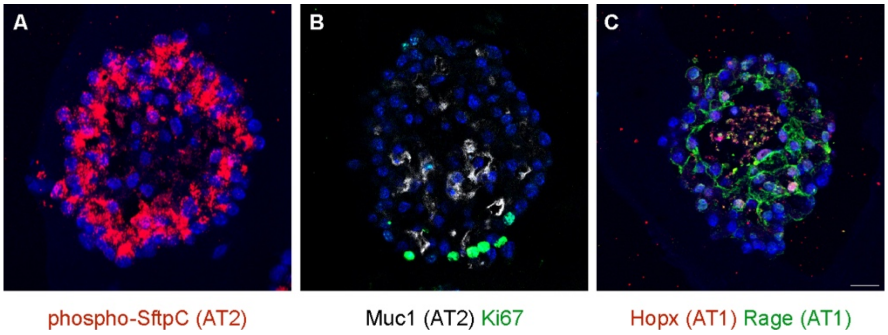

Fig. S1. Immunolabeling of alveolospheres.
(A-C) Alveolospheres immunolabelled for epithelial markers. Scale bar, 10 μm.

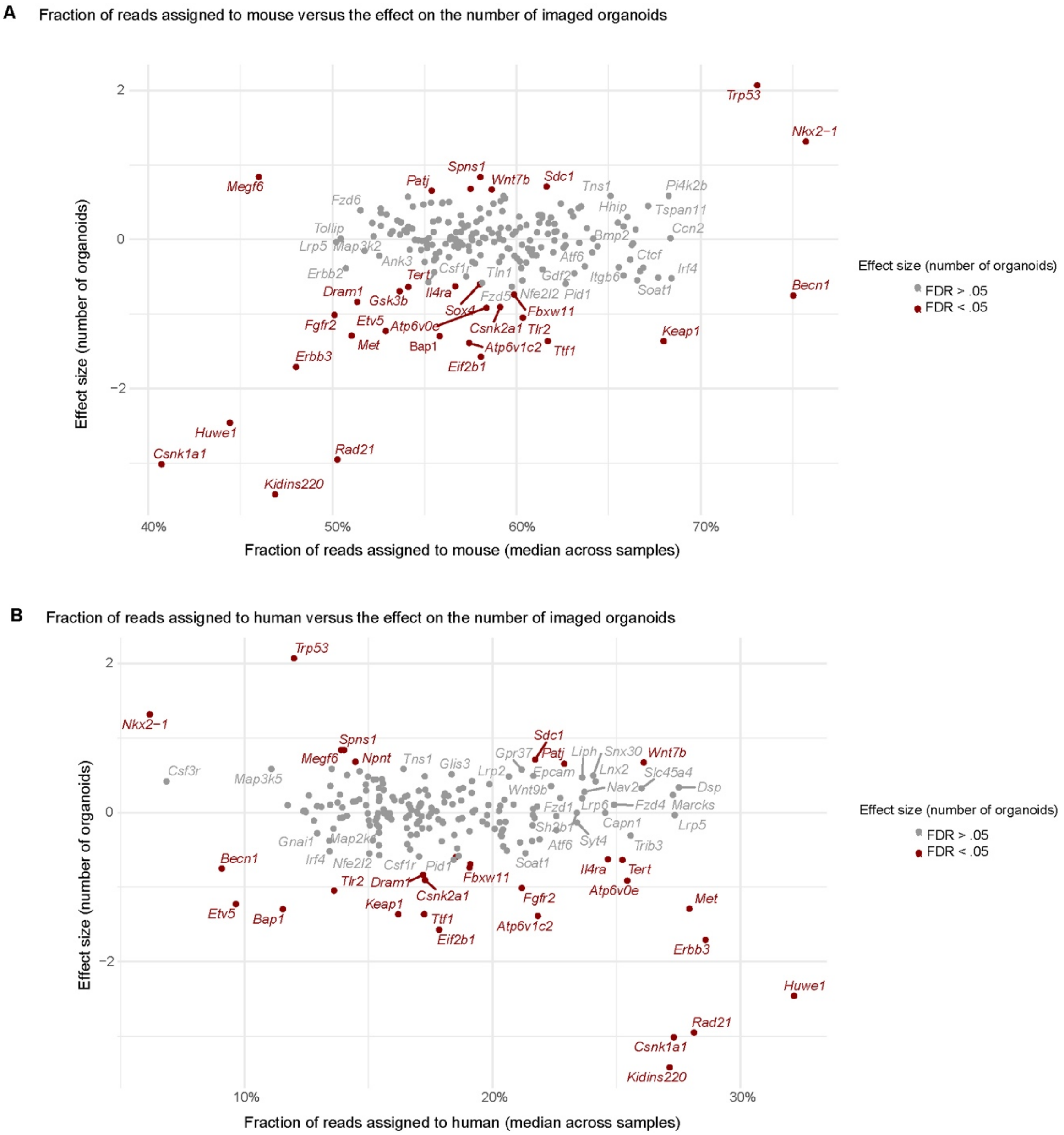

Fig. S2. The fraction of reads assigned to the mouse transcriptome scales with alveolosphere growth.
For each gene knockout (KO), the fraction of total RNAseq reads assigned to the mouse (A) or human (B) genomes (median across samples) is compared to the effect size of the KO on the number of alveolospheres per well relative to the in-plate *Tigit* sgRNA control (n=4 wells per knockout). Knockouts with a significant effect on the number of alveolospheres (FDR<0.05) relative to the in-plate *Tigit* sgRNA control shown in red.

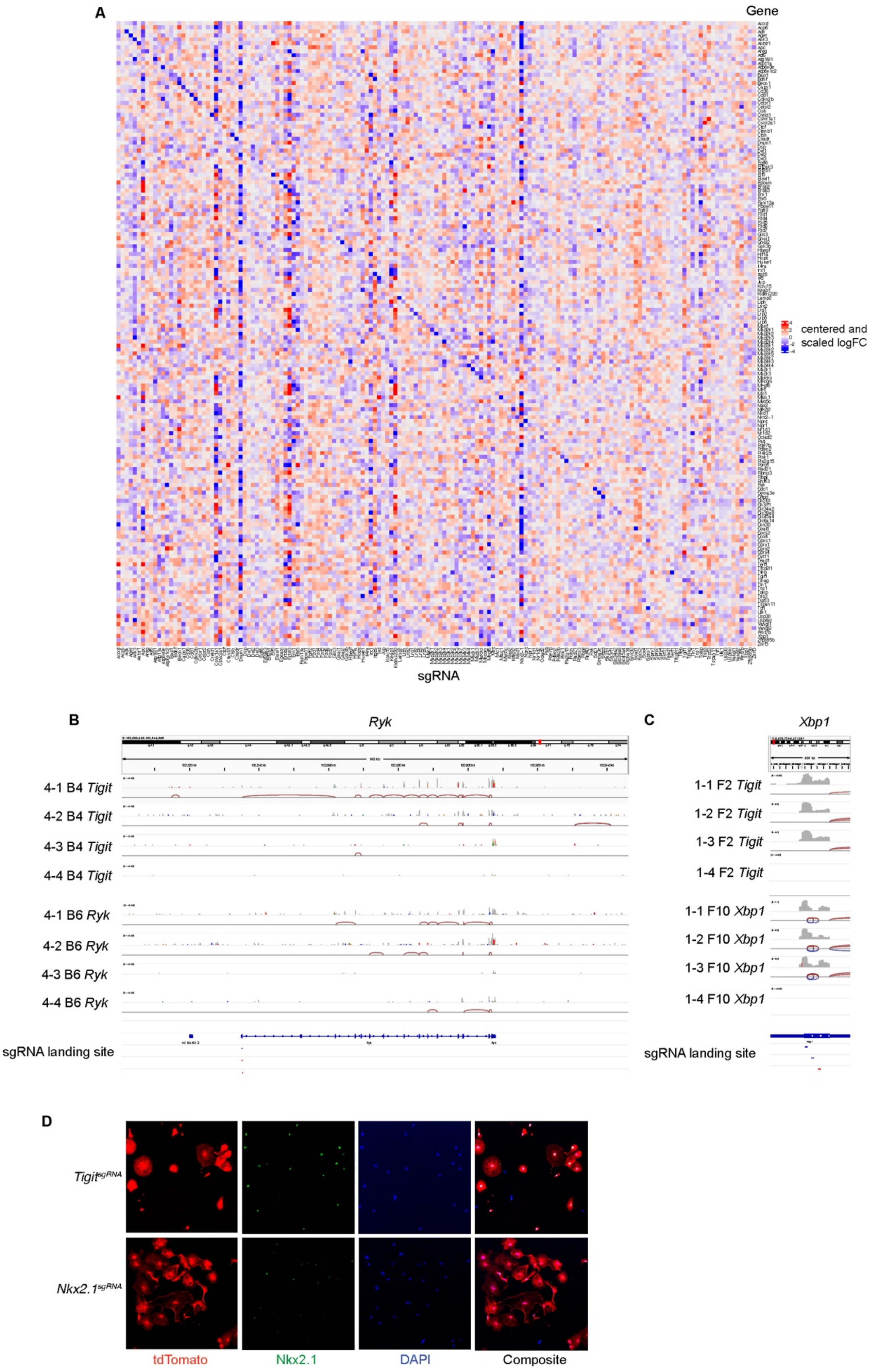
A
Gene
centered and
scaled logFC
sgRNA
B
Ryk
4-1 B4 Tigit
4-2 B4 Tigit
4-3 B4 Tigit
4-4 B4 Tigit
4-1 B6 Ryk
4-2 B6 Ryk
4-3 B6 Ryk
4-4 B6 Ryk
sgRNA landing site
C
Xbp1
1-1 F2 Tigit
1-2 F2 Tigit
1-3 F2 Tigit
1-4 F2 Tigit
1-1 F10 Xbp1
1-2 F10 Xbp1
1-3 F10 Xbp1
1-4 F10 Xbp1
sgRNA landing site
D
Tigit sgRNA
Nkx2.1 sgRNA
tdTomato
Nkx2.1
DAPI
Composite

Fig. S3. Determining gene knockout efficiency in AT2 cells.
(A) Heatmap depicts differential expression (Log2 fold change) of each target gene compared with the *Tigit* and *tdTomato*-deleted control wells across the different knockouts. (B, C) The location and number of murine *Ryk* (B) or *Xbp1* (C) reads in alveolospheres after CRISPR gene editing with the indicated sgRNAs (n=4). The sgRNA landing sites within each gene are indicated (bottom). Samples are labeled as 'Plate-Replicate Well sgRNA' (e.g., 1-1 F2 Tigit indicates plate 1, replicate 1, well F2 received *Tigit* targeting RNPs). Loops within the targeted exon (bottom) depict cryptic junctions that correspond to deletions. Note that samples from plate 4 have low *Xbp1* expression. (D) Lineage-labeled AT2 cells (red) immunolabeled for Nkx2.1 (green) and stained with DAPI (blue) at 3 days after Cas9-RNP nucleofection with the indicated guides. Scale bar, 10 μM.

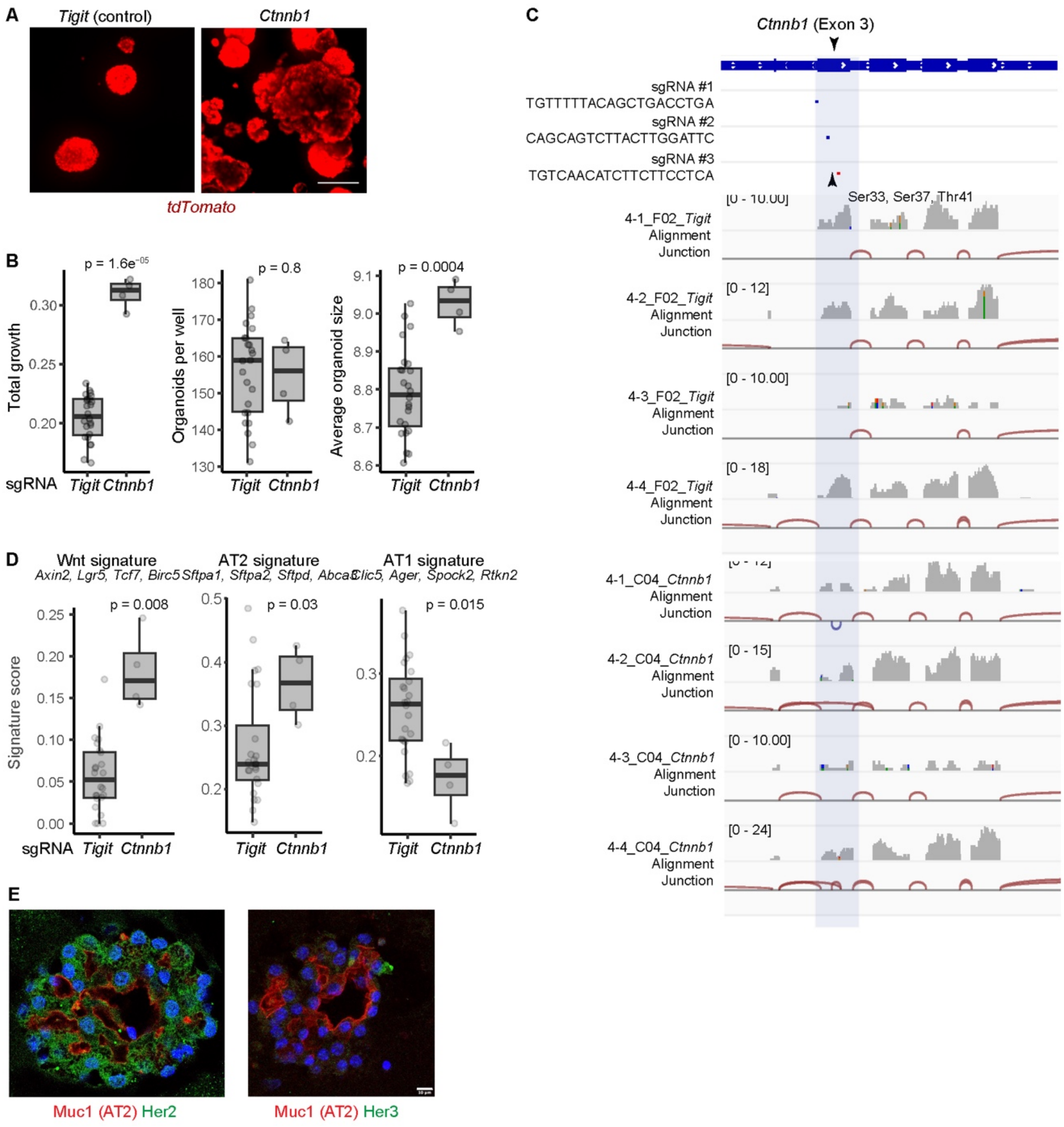

Fig. S4. Characterization of *Ctnnb1* mutant alveolospheres.
(A) TdTomato+ alveolospheres from *Tigit* and *Ctnnb1* mutant AT2 cells. Scale bar, 500 µm. (B) The size (left), number (center), and total growth (right; tdTomato area/area of well) of the alveolospheres in (A). n=4 replicates for *Ctnnb1,* n=24 for the in-plate *Tigit* control. (C) *Ctnnb1* reads after RNAseq of *Tigit* and *Ctnnb1* mutant alveolospheres are aligned to the mouse reference genome, labeled as in Fig. S3C. Loops between exons show cryptic junction reads that skip over exon 3. (D) Expression of the indicated gene signatures (see methods) of $Ctnnb1^{KO}$ (n=4) and in-plate *Tigit* control (n=24) alveolospheres. P values determined by 2-sided student t test. (E) Alveolospheres immunolabelled for AT2 marker Muc1 (red) and Her2 (green). Scale bar, 10 µm.

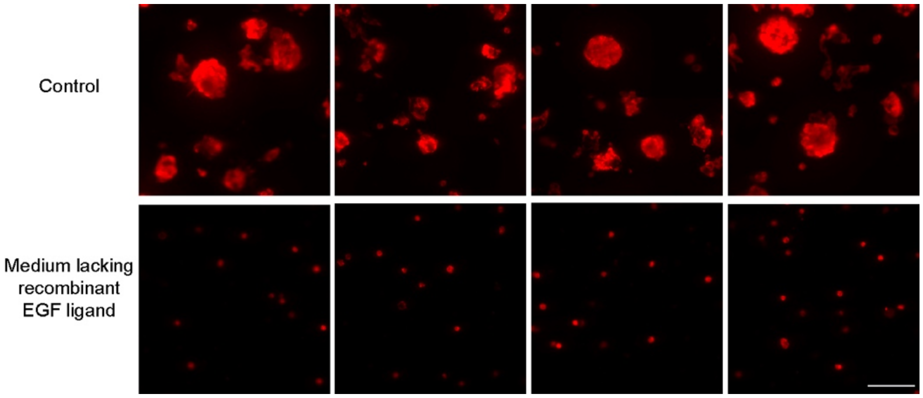

Fig. S5. EGF is required for alveolosphere growth.
tdTomato$^+$ alveolospheres grown in regular medium (top) or medium lacking recombinant EGF (bottom). Four replicate wells are shown. Scale bar, 500 μm.

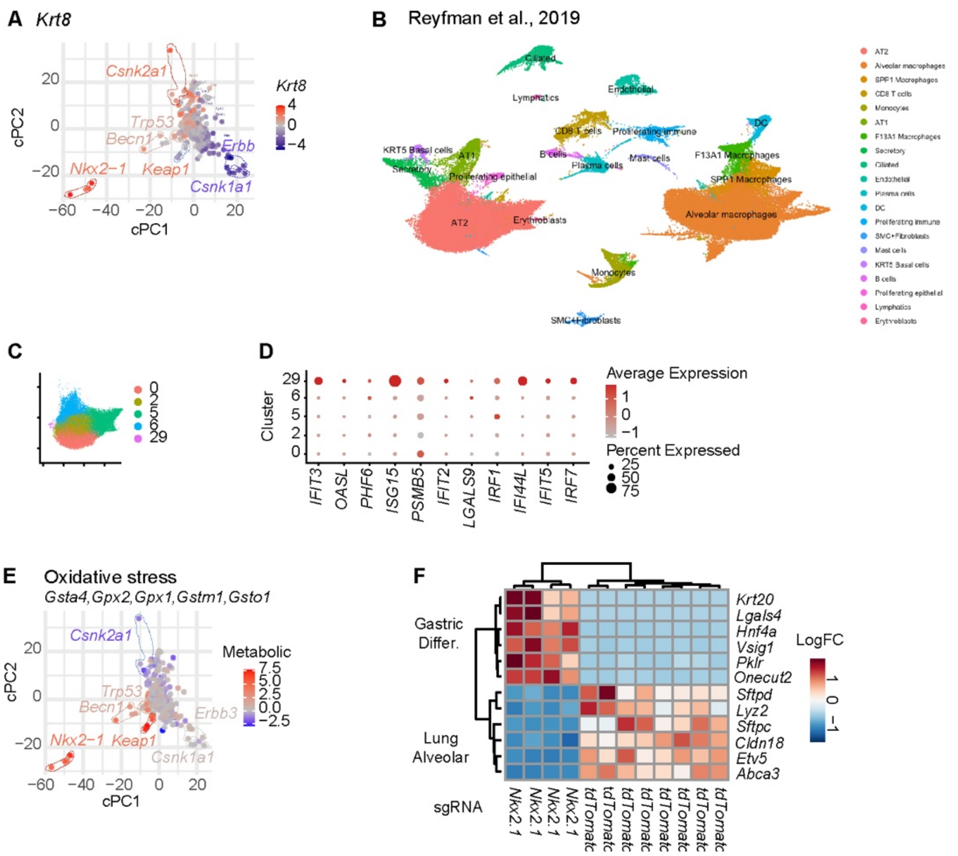

Fig. S6. Single cell RNAseq data from normal and diseased human lungs reveals a population of AT2 cells expressing an interferon gene signature.

(A) Two-dimensional cPCA representation of the murine AT2 transcriptional space of the alveolospheres in Fig. 3A. Each dot represents the transcriptome from a single replicate of the indicated knockout. Dot color indicates differential expression of *Krt8* (A, log2FC). (B) Two-dimensional PCA representation of the human lung single cell RNAseq data as calculated in Reyfman et al., 2019 (16). (C) AT2 subsets from (A) defined by author's PCA analysis (16). (D) Expression of a selection of interferon-stimulated genes by the AT2 subsets in (B). Red coloring indicates the mean level of gene expression and dot size the percentage of positive cells. (E) Two-dimensional cPCA representation of the murine AT2 transcriptional space of the alveolospheres in Fig. 3A. Each dot represents the transcriptome from a single replicate of the indicated knockout. Dot color indicates the scaled expression of the indicated gene signature. (F) Heatmap compares expression (Log CPM) of alveolar and gastric markers (rows) across replicates of *Nkx2.1*^{KO} and *tdTomato*^{KO} control alveolospheres (columns).

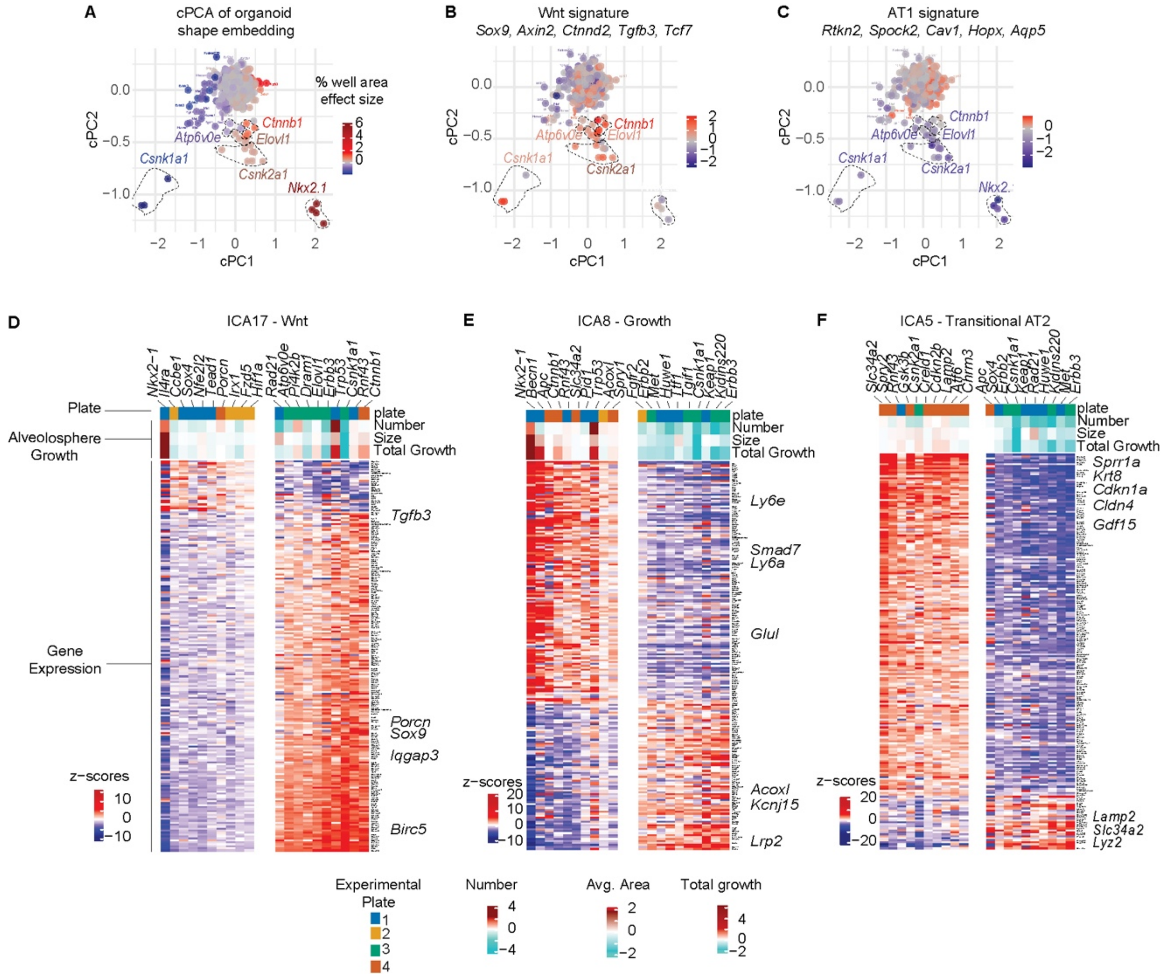

Fig. S7. Independent Component Analysis of the AT2 cell transcriptome.
(A-C) cPCA of the Segment-Anything embeddings from segmented alveolosphere regions, averaged per well. Each dot represents a well with the specified gene knockout, plotted in the space defined by cPC1 and cPC2. The color of each dot represents expression of the indicated gene signature (B-C; see methods). (D-E) Heatmaps of the 200 genes (rows) and 20 knockouts (columns) with the highest contribution to the indicated independent components ranked according to their contribution. Above the gene expression data, the heatmap is annotated with the alveolosphere growth (Imaging data; Green-to-brown heatmap) and experimental plate of origin (multi color) for each knockout.

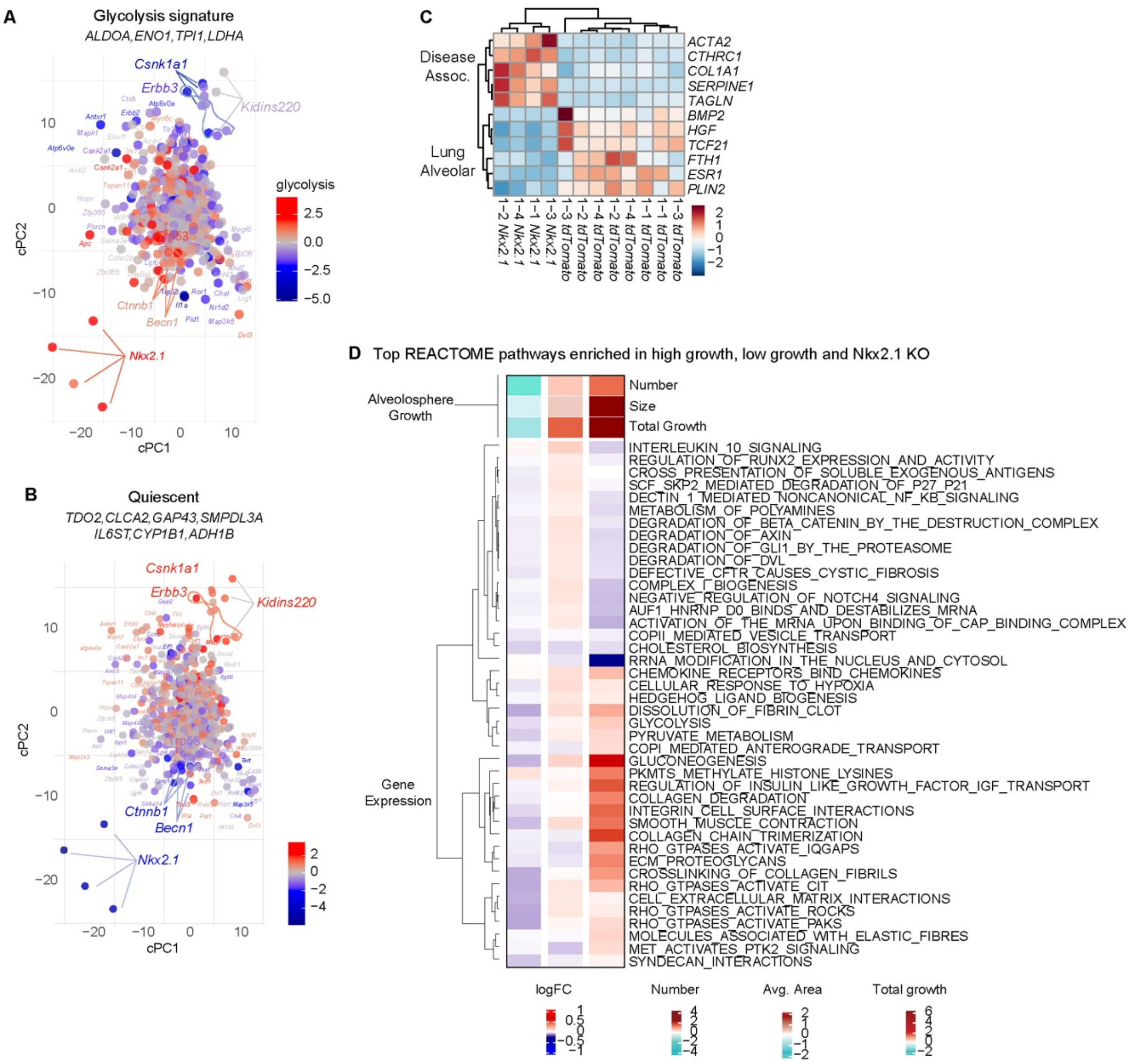

Fig. S8. AT2 cells shape their fibroblast niche.
(A-B) cPCA of the transcriptomic profiles of human fibroblasts in chimeric alveolospheres (based on the top 3,000 highly variable genes). Dots represent the human transcriptome from individual gene knockout replicates plotted in the space defined by contrastive PC1 (cPC1) and contrastive PC2 (cPC2). Dot color shows expression of the indicated gene signature. (C) Heatmaps show differential expression of fibroblast markers (LogFC) in *Nkx2.1*^{KO} and *tdTomato*^{KO} alveolospheres. (D) Heatmaps show average log2-fold-changes of the top Reactome gene sets (adjusted p-value < 1%) in low growth (left) versus high growth (middle) growth as measured by imaging (green-to-brown heatmap, top) and how their expression changes in *Nkx2.1*^{KO} wells (right).

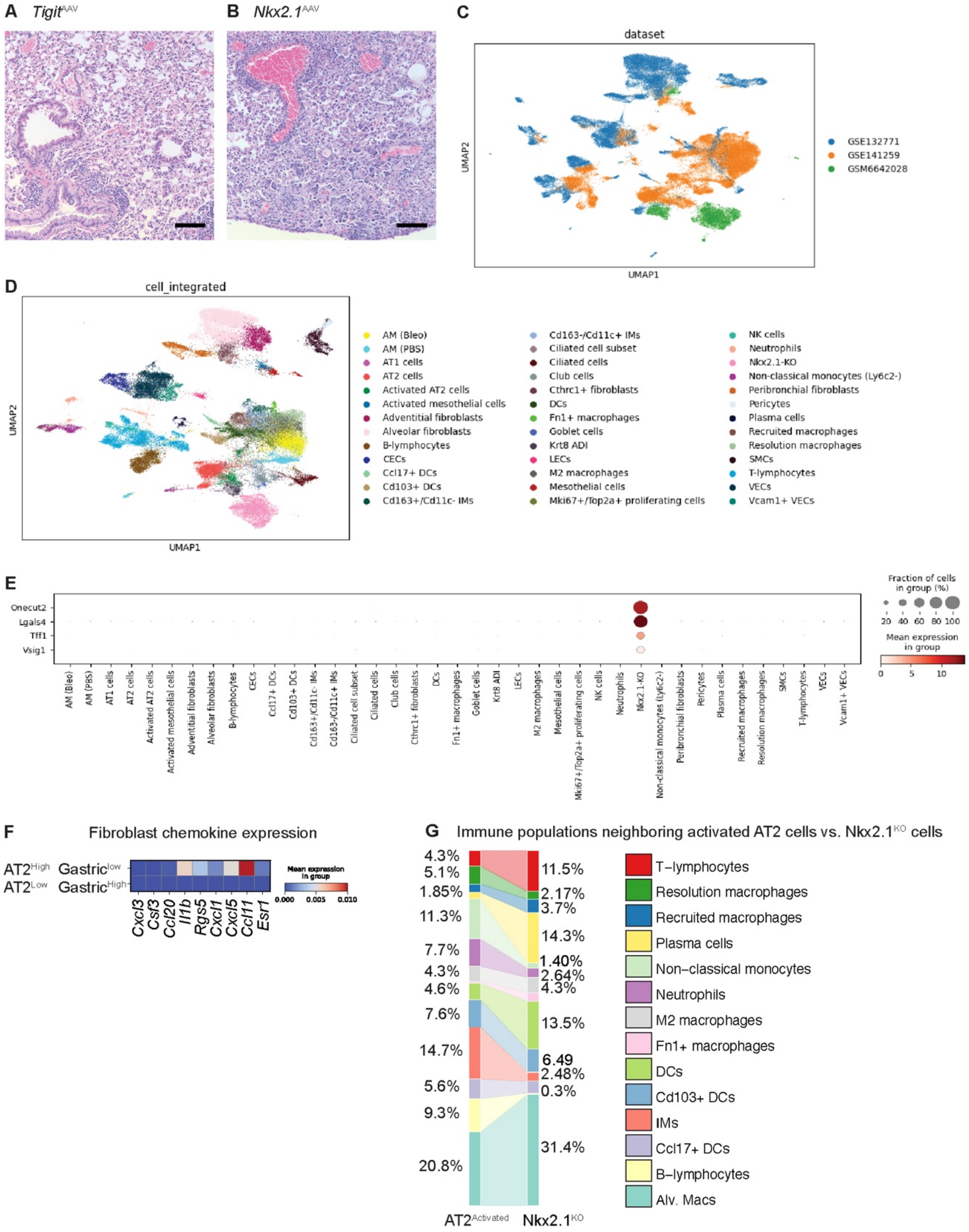

Fig. S9. Analyzing the effect of viral *Nkx2.1*^{KO} with spatial transcriptomics.
(A-B) Representative micrographs of H&E sections of lungs from *Tigit*^{AAV} (A) and *Nkx2.1*^{AAV} (B) mice. Scale bars, 100 μm. (C-D) UMAP of the transcriptomic profiles of lung reference atlas derived from the indicated datasets in (C). Panel (D) displays the cell types captured within the reference atlas after harmonization. (E) The mean level of gene expression (dot intensity, red scale) and the percentage of positive cells (dot size) determined by single cell RNAseq in harmonized datasets from (C). (F) Heatmap displays fibroblast expression (Log CPM) of the

indicated chemokines within AT2^{high} or Gastric^{high} regions in spatial data. (G) Composition plots displaying the relative abundance of immune cells neighboring activated AT2 versus *Nkx2.1*^{KO} cells.

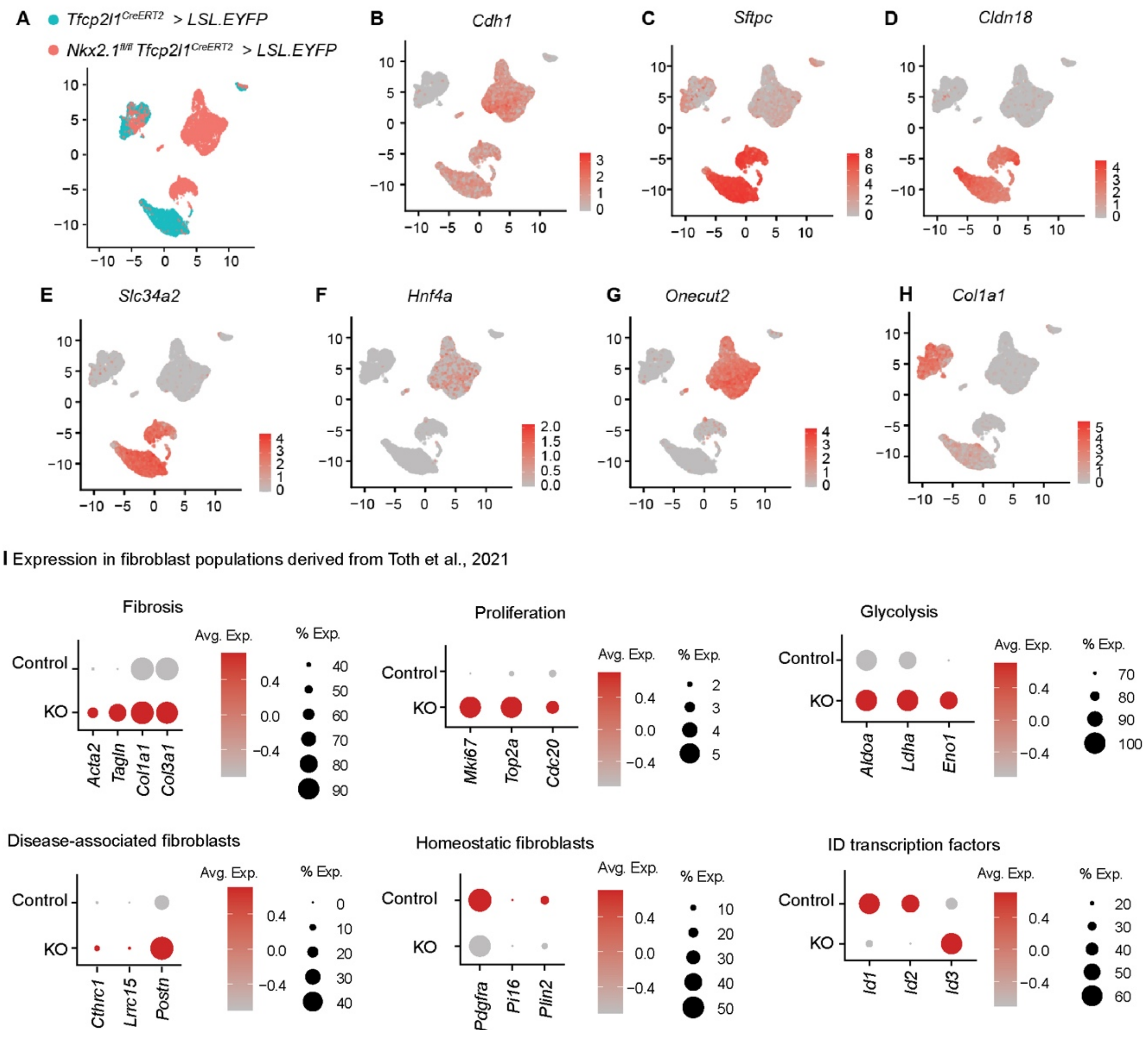

Fig. S10. Analyzing the cell autonomous and non-cell autonomous effect of *Nkx2.1^{KO}* using mouse genetics.

(A) PCA representation of mouse lung single cell RNAseq data from Toth et al., 2023 (72). Dot color depicts whether cells derive from control (teal) or *Nkx2.1^{cKO}* lungs (red). (B-H) PCA representations of the data in (A) with dot color representing log(Counts) of the genes indicated. (I) Dot plots showing gene expression in *Col1a1^{+}* fibroblasts from control or *Nkx2.1^{cKO}* lungs in (F). Dot color indicates the mean level of scaled gene expression and dot size the percentage of positive cells.

SI References

1. Desai, T.J., Brownfield, D.G., and Krasnow, M.A. (2014). Alveolar progenitor and stem cells in lung development, renewal and cancer. Nature *507*, 190–194. https://doi.org/10.1038/nature12930.
2. Kirillov, A., Mintun, E., Ravi, N., Mao, H., Rolland, C., Gustafson, L., Xiao, T., Whitehead, S., Berg, A.C., Lo, W.-Y., et al. (2023). Segment Anything. arXiv. https://doi.org/10.48550/arxiv.2304.02643.
3. Moser, N.H., Landauer, A.K., and Kafka, O.L. (2025). IMPPY3D: Image Processing in Python for 3D Image Stacks. J. open source Softw. *10*, 10.21105/joss.07405. https://doi.org/10.21105/joss.07405.
4. https://app.readthedocs.org/projects/pillow/downloads/pdf/latest/.
5. Abid, A., Zhang, M.J., Bagaria, V.K., and Zou, J. (2018). Exploring patterns enriched in a dataset with contrastive principal component analysis. Nat. Commun. *9*, 2134. https://doi.org/10.1038/s41467-018-04608-8.
6. Hagemann-Jensen, M., Ziegenhain, C., Chen, P., Ramsköld, D., Hendriks, G.-J., Larsson, A.J.M., Faridani, O.R., and Sandberg, R. (2020). Single-cell RNA counting at allele and isoform resolution using Smart-seq3. Nat. Biotechnol. *38*, 708–714. https://doi.org/10.1038/s41587-020-0497-0.
7. Conway, T., Wazny, J., Bromage, A., Tymms, M., Sooraj, D., Williams, E.D., and Beresford-Smith, B. (2012). Xenome--a tool for classifying reads from xenograft samples. Bioinform. (Oxf., Engl.) *28*, i172-8. https://doi.org/10.1093/bioinformatics/bts236.
8. https://bioconductor.org/packages/release/bioc/html/HTSeqGenie.html.
9. Wu, T.D., and Nacu, S. (2010). Fast and SNP-tolerant detection of complex variants and splicing in short reads. Bioinformatics *26*, 873–881. https://doi.org/10.1093/bioinformatics/btq057.
10. Wu, T.D., Reeder, J., Lawrence, M., Becker, G., and Brauer, M.J. (2016). Statistical Genomics. Methods Mol. Biol. *1418*, 283–334. https://doi.org/10.1007/978-1-4939-3578-9_15.
11. Robinson, M.D., and Oshlack, A. (2010). A scaling normalization method for differential expression analysis of RNA-seq data. Genome Biol. *11*, R25–R25. https://doi.org/10.1186/gb-2010-11-3-r25.
12. Boileau, P., Hejazi, N., and Dudoit, S. (2020). scPCA: A toolbox for sparse contrastive principal component analysis in R. J. Open Source Softw. *5*, 2079. https://doi.org/10.21105/joss.02079.
13. Lun, A.T.L., McCarthy, D.J., and Marioni, J.C. (2016). A step-by-step workflow for low-level analysis of single-cell RNA-seq data with Bioconductor. F1000Research *5*, 2122. https://doi.org/10.12688/f1000research.9501.2.
14. Ritchie, M.E., Phipson, B., Wu, D., Hu, Y., Law, C.W., Shi, W., and Smyth, G.K. (2015). limma powers differential expression analyses for RNA-sequencing and microarray studies. Nucleic Acids Res. *43*, e47–e47. https://doi.org/10.1093/nar/gkv007.
15. Law, C.W., Chen, Y., Shi, W., and Smyth, G.K. (2014). voom: precision weights unlock linear model analysis tools for RNA-seq read counts. Genome Biol. *15*, R29–R29. https://doi.org/10.1186/gb-2014-15-2-r29.
16. Wu, D., and Smyth, G.K. (2012). Camera: a competitive gene set test accounting for inter-gene correlation. Nucleic Acids Res. *40*, e133–e133. https://doi.org/10.1093/nar/gks461.
17. Cardoso, J.F., and Souloumiac, A. (1993). Blind beamforming for non-gaussian signals. IEE Proc. F Radar Signal Process. *140*, 362. https://doi.org/10.1049/ip-f-2.1993.0054.
18. https://cran.r-project.org/web/packages/ica/index.html.
19. Strunz, M., Simon, L.M., Ansari, M., Kathiriya, J.J., Angelidis, I., Mayr, C.H., Tsidiridis, G., Lange, M., Mattner, L.F., Yee, M., et al. (2020). Alveolar regeneration through a Krt8+ transitional stem cell state that persists in human lung fibrosis. Nat. Commun. *11*, 3559. https://doi.org/10.1038/s41467-020-17358-3.
20. Tsukui, T., Sun, K.-H., Wetter, J.B., Wilson-Kanamori, J.R., Hazelwood, L.A., Henderson, N.C., Adams, T.S., Schupp, J.C., Poli, S.D., Rosas, I.O., et al. (2020). Collagen-producing lung cell atlas identifies multiple subsets with distinct localization and relevance to fibrosis. Nat. Commun. *11*, 1920. https://doi.org/10.1038/s41467-020-15647-5.

21. Toth, A., Kannan, P., Snowball, J., Kofron, M., Wayman, J.A., Bridges, J.P., Miraldi, E.R., Swarr, D., and Zacharias, W.J. (2023). Alveolar epithelial progenitor cells require Nkx2-1 to maintain progenitor-specific epigenomic state during lung homeostasis and regeneration. Nat. Commun. *14*, 8452. https://doi.org/10.1038/s41467-023-44184-0.
22. Gayoso, A., Lopez, R., Xing, G., Boyeau, P., Amiri, V.V.P., Hong, J., Wu, K., Jayasuriya, M., Mehlman, E., Langevin, M., et al. (2022). A Python library for probabilistic analysis of single-cell omics data. Nat. Biotechnol. *40*, 163–166. https://doi.org/10.1038/s41587-021-01206-w.
23. Cable, D.M., Murray, E., Zou, L.S., Goeva, A., Macosko, E.Z., Chen, F., and Irizarry, R.A. (2022). Robust decomposition of cell type mixtures in spatial transcriptomics. Nat. Biotechnol. *40*, 517–526. https://doi.org/10.1038/s41587-021-00830-w.
